# Computational neuroanatomy of human stratum proprium of interparietal sulcus

**DOI:** 10.1007/s00429-017-1492-1

**Published:** 2017-09-04

**Authors:** Maiko Uesaki, Hiromasa Takemura, Hiroshi Ashida

**Affiliations:** 1 Department of Psychology, Graduate School of Letters, Kyoto University, Kyoto, Japan; 2 Japan Society for the Promotion of Science, Tokyo, Japan; 3 Open Innovation and Collaboration Research Organization, Ritsumeikan University, Osaka, Japan; 4 Center for Information and Neural Networks (CiNet), National Institute of Information and Communications Technology, and Osaka University, Suita, Japan; 5 Graduate School of Frontier Biosciences, Osaka University, Suita, Japan

**Keywords:** Stratum proprium of interparietal sulcus, Diffusion-weighted MRI, Tractography, fMRI, Optic flow, Visuo-vestibular integration

## Abstract

Recent advances in diffusion-weighted MRI (dMRI) and tractography have enabled identification of major long-range white matter tracts in the human brain. Yet, our understanding of shorter tracts, such as those within the parietal lobe, remains limited. Over a century ago, a tract connecting the superior and inferior parts of the parietal cortex was identified in a post-mortem study: stratum proprium of interparietal sulcus (SIPS; Sachs, Das hemisphärenmark des menschlichen grosshirns. Verlag von georg thieme, Leipzig, 1892). The tract has since been replicated in another fibre dissection study (Vergani et al., Cortex 56:145–156, 2014), however, it has not been fully investigated in the living human brain and its precise anatomical properties are yet to be described. We used dMRI and tractography to identify and characterise SIPS in vivo, and explored its spatial proximity to the cortical areas associated with optic-flow processing using fMRI. SIPS was identified bilaterally in all subjects, and its anatomical position and trajectory are consistent with previous post-mortem studies. Subsequent evaluation of the tractography results using the linear fascicle evaluation and virtual lesion analysis yielded strong statistical evidence for SIPS. We also found that the SIPS endpoints are adjacent to the optic-flow selective areas. In sum, we show that SIPS is a short-range tract connecting the superior and inferior parts of the parietal cortex, wrapping around the intraparietal sulcus, and that it may be a crucial anatomy underlying optic-flow processing. In vivo identification and characterisation of SIPS will facilitate further research on SIPS in relation to cortical functions, their development, and diseases that affect them.

**Electronic supplementary material:**

The online version of this article (doi:10.1007/s00429-017-1492-1) contains supplementary material, which is available to authorised users.

## Introduction

Anatomical connections through the white matter axon bundles (i.e. fascicles, tracts) establish fundamental features of the brain’s information processing (Catani and Ffytche 2005; Catani and Thiebaut de Schotten 2012; Bullock et al. 2005; Fields 2008, 2015; Wandell and Yeatman 2013; Wandell 2016). Diffusion-weighted magnetic resonance imaging (dMRI) and tractography provide a unique opportunity to identify and characterise the white matter tracts in the living human brain (Catani et al. 2002; Wakana et al. 2004; Mori and Zhang 2006; Catani and Thiebaut de Schotten 2012; Craddock et al. 2013; Wandell 2016; Rokem et al. 2017).

A body of dMRI research has successfully identified several major long-range white matter tracts, such as the superior longitudinal fasciculus and the inferior longitudinal fasciculus, in a consistent manner with the known post-mortem anatomy (Catani et al. 2002; Wakana et al. 2004; Schmahmann et al. 2007), and ergo opened new avenues to research on the properties of major human white matter tracts in relation to development and diseases (Lebel et al. 2012; Yeatman et al. 2012a, 2014a; Ogawa et al. 2014; Malania et al. 2017). More recent dMRI studies have identified shorter white matter tracts including frontal aslant tract and vertical occipital fasciculus, which, partially for their relatively short trajectories, had previously received very little attention in the neuroscience literature (Thiebaut de Schotten et al. 2012; Catani and Thiebaut de Schotten 2012; Catani et al. 2013; Yeatman et al. 2013, 2014b; Takemura et al. 2016b, 2017). Some of those studies have suggested the importance of the shorter tracts in relation to cognitive functions and diseases (Kinoshita et al. 2015; Kemerdere et al. 2016; Kronfeld-Duenias et al. 2016; Duan et al. 2015; Takemura et al. 2016b; Lee Masson et al. 2017).

Here, we focus on a short white matter tract connecting the superior and inferior parts of the parietal cortex, wrapping around the inferior parietal sulcus. This tract was initially described by the German neurologist Heinrich Sachs (1892) as the stratum proprium of interparietal sulcus (hereafter, we refer to this tract as SIPS). Except for one recent fibre dissection study replicating Sachs’s work (Vergani et al. 2014), this tract has been largely overlooked in the literature. Given the functional MRI (fMRI) evidence indicating the involvement of the superior and inferior parts of the parietal cortex in crucial cognitive functions (Corbetta and Shulman 2002; Culham et al. 2006; Uncapher and Wagner 2009; Cardin and Smith 2010; Blanke 2012; Greenlee et al. 2016), SIPS is likely a necessary and important tract supporting those functions. Yet, the characteristics of SIPS are poorly understood due to the lack of studies replicating SIPS in the living human brain, using three-dimensional digital anatomical data such as dMRI and reproducible computational analyses.

One of the cortical functions that involve the parietal cortex is optic-flow processing. Optic flow is the pattern of visual motion signals elicited by self-motion (Gibson 1950, 1954), and is an important cue to accurate perception of self-motion. A network of sensory areas in the parietal cortex has been shown to be involved in optic-flow processing (Cardin and Smith 2010, 2011). Those optic-flow selective areas include the ventral intraparietal area (VIP), the precuneus motion area (PcM) and the putative area 2v (p2v) located in the superior part of the parietal cortex, and the posterior-insular complex (PIC+: Deutschländer et al. 2004; Cardin and Smith 2010, 2011; Biagi et al. 2015; Uesaki and Ashida 2015; Wada et al. 2016) in the inferior part of the parietal cortex. Several studies have described a convergence of visual and vestibular information regarding self-motion, involving those optic-flow selective areas (Kleinschmidt et al. 2002; Wiest et al. 2004; Kovács et al. 2008; Fetsch et al. 2009; Butler et al. 2010; Prsa et al. 2015; Uesaki and Ashida 2015). To fully understand the underlying mechanism of optic-flow processing, it is essential to investigate how communication between the superior and inferior parts of the parietal cortex is supported by the white matter anatomy.

We used dMRI and tractography to identify the anatomical location and trajectory of SIPS in the living human brain. The ensemble tractography (Takemura et al. 2016a) yielded bilateral identification of SIPS in all subjects. Evidence for SIPS was evaluated based on the consistency across datasets, comparison with post-mortem fibre dissection studies (Sachs 1892; Vergani et al. 2014), and the virtual lesion analysis (Pestilli et al. 2014; Leong et al. 2016; Gomez et al. 2015; Takemura et al. 2016b). We also explored the functional relevance of SIPS by performing fMRI experiments on the same subjects and examining the spatial proximity between the SIPS endpoints and functionally defined cortical regions previously associated with optic-flow processing (Cardin and Smith, 2010, 2011; Greenlee et al. 2016). Results showed that the dorso-lateral SIPS endpoints are near VIP, PcM and p2v, whereas the ventro-medial SIPS endpoints are near PIC+; placing SIPS in a good position to channel sensory signals between the distant cortical areas underlying visuo-vestibular integration necessary for optic-flow processing and perception of self-motion. Finally, we also demonstrate evidence of SIPS in additional 90 subjects from publically available Human Connectome Project datasets.

For the first time in the living human brain, we describe human SPIS in a manner consistent with the previous post-mortem dissection studies. Our findings confirm that SIPS is a short-range tract connecting the superior and inferior parts of the parietal cortex, wrapping around the intraparietal sulcus. The findings also highlight that in vivo identification and characterisation of SIPS open new avenues to studying this tract in relation to cortical functions, their development, and diseases that affect them.

To facilitate future research, we make the code to identify human SIPS publicly available in Github repository [https://github.com/htakemur/SIPS] and also as part of AFQ toolbox [Yeatman et al. 2012b; https://github.com/yeatmanlab/AFQ].

## Materials and methods

We analysed dMRI data of 100 human subjects, from three independent datasets. One set of dMRI data was acquired at Kokoro Research Center, Kyoto University (KU dataset), along with fMRI measurements to identify cortical regions activated by optic-flow stimulation. The other dMRI datasets were obtained from the Human Connectome Project (HCP) by WU-Minn Consortium (WU-Minn HCP; Van Essen et al. 2013) and MGH-USC Consortium (MGH-USC HCP; Fan et al. 2016).

### Subjects: KU dataset

Six healthy volunteers (three males and three females; of the ages between 22 and 47 years; subjects S1–S6) participated in the study. All six subjects underwent both dMRI and fMRI experiments. All had normal or corrected-to-normal vision. All individual subjects gave written informed consent to take part in this study, which was conducted in accordance with the ethical standards stated in the Declaration of Helsinki and approved by the local ethics and safety committees at Kyoto University.

### Data acquisition and preprocessing: KU dataset

All MR images were obtained with a 3-T Siemens Magnetom Verio scanner (Siemens, Erlangen, Germany), using a Siemens 32-channel head coil, at Kokoro Research Center, Kyoto University.

#### Diffusion-weighted MRI data

Two repeated acquisitions of MR images were conducted for each subject, using a diffusion-weighted single-shot spin echo, echo-planar sequence [60 axial slices, 2-mm isotropic voxels, time of repetition (TR) 8300 ms, time echo (TE) 94 ms, field of view (FoV) 200 × 200 mm^2^]. The dMRI data were sampled in 64 directions with a *b* value of 1000 s/mm^2^. Two non-diffusion-weighted images (*b* = 0) were obtained.

Diffusion data were preprocessed using mrDiffusion, implemented in Matlab as part of the mrVista software distribution (https://github.com/vistalab/vistasoft). MR images in each scan were motion-corrected to the *b* = 0 image acquired in the same scan, using a rigid-body alignment algorithm implemented in SPM (Friston and Ashburner 2004). Eddy current correction and head-motion correction were applied in the process of 14-parameter constrained nonlinear coregistration, based on the expected patterns of eddy current distortion given the phase-encoding directions of the acquired data (Rohde et al. 2004). The gradient direction in each diffusion-weighted volume was corrected using the rotation parameters from the motion correction procedure. Subsequently, fibre orientation in each voxel was estimated using constrained spherical deconvolution (CSD; Tournier et al. 2007; *L*_max_ = 8) implemented in MRtrix (Tournier et al. 2012). CSD allows for tractography based on a model that is capable of accounting for crossing fibres. Because the tract connecting the superior and inferior parts of the parietal cortex likely intersects with the neighbouring major fasciculi such as the arcuate fasciculus, CSD was employed to fully reconstruct the tract.

#### T1-weighted image data

For each subject, a high-resolution T1-weighted 3D anatomical image was acquired with a magnetisation-prepared rapid-acquisition gradient echo (MP-RAGE; 208 axial slices, 1-mm isotropic voxels, TR 2250 ms, TE 3.51 ms, FoV 256 × 256 mm^2^, flip angle 9°, bandwidth 230 Hz/pixel), and the border between the grey matter and white matter was defined. Initial segmentation was performed using an automated procedure in Freesurfer (Fischl 2012), which was then refined manually (Yushkevich et al. 2006).

#### Functional data and localisation of optic-flow selective sensory regions

To determine the spatial relations between the white matter tract and the optic-flow selective sensory areas (Cardin and Smith 2011; Frank et al. 2014), four regions of interest (ROIs) were identified with data acquired in separate fMRI localiser scans, using the procedure described in Uesaki and Ashida (2015). The stimulus was a random dot kinematogram consisting of 200 moving light dots of 10 × 10 pixels (subtending approximately 0.4° × 0.4° visual angle) on a dark background. The dots initially appeared at random locations and formed a circular patch of 30° diameter. Motion directions of the dots were arranged so that the dots (a) cycled through a spiral space with time-varying trajectories away from and towards the centre of the patch for the optic-flow stimulus, or (b) moved in random directions for the random-motion stimulus. The coherent optic-flow and random-motion stimuli were presented in 16-s blocks. Subjects maintained central fixation throughout the experiment. No attentional task was undertaken.

Functional data were acquired with a gradient echo, echo-planar sequence (39 contiguous axial slices, 3-mm isotropic voxels, TR 2000 ms, TE 25 ms, FoV 192 × 192 mm^2^, flip angle 80°, bandwidth 2368 Hz/pixel), using a Siemens 32-channel posterior-head array coil; which gave an improved signal-to-noise ratio in the occipital cortex at the expense of the anterior part of the brain. Each subject underwent two fMRI scans. Functional data were then preprocessed and analysed with BrainVoyager QX (version 2.6, Brain Innovation, Maastricht, The Netherlands). Analysis was conducted by fitting a general linear model (GLM). Each of the 16-s stimulus blocks was convolved with a canonical haemodynamic response function, and entered into a multiple-regression analysis to generate parameter estimates for each regressor at each voxel. Blood-oxygen-level-dependent responses to the coherent optic-flow and random-motion stimuli were contrasted, which allowed for isolation of cortical regions that are significantly more sensitive to coherent optic flow at *p* value (uncorrected) of less than 0.005. The analysis was performed on the 3D anatomical volumes for each subject. The ROIs were defined as all contiguous voxels that were significantly more active with coherent optic-flow stimulation than with random-motion stimulation (Uesaki and Ashida 2015); in the ventral intra-parietal cortex (VIP), the precuneus motion area (PcM), the junction of the intra-parietal sulcus (p2v), and the posterior region of the insular cortex (PIC+). Stimulus design and analysis methods are described in more detail elsewhere (Uesaki and Ashida 2015).

### Data acquisition and preprocessing: Human Connectome Project datasets

Diffusion-weighted MRI data obtained from 61 subjects in WU-Minn HCP dataset (S7–S67: Van Essen et al. 2013) and 33 subjects in MGH-USC HCP dataset (S68–S100: Fan et al. 2016) were also analysed in this study. These data were acquired with multiple *b* values (1000, 2000 and 3000 s/mm^2^ for WU-Minn HCP dataset; 1000, 3000, 5000 and 10,000 s/mm^2^ for MGH-USC HCP dataset), high spatial (1.25 × 1.25 × 1.25 mm^3^ for WU-Minn HCP dataset; 1.5 × 1.5 × 1.5 mm^3^ for MGH-USC HCP dataset) and angular resolution (90 directions at each *b* value for WU-Minn HCP dataset; 64 directions at *b* = 1000, 3000 s/mm^2^ and 128 directions at *b* = 5000, 10,000 s/mm^2^ for MGH-USC HCP dataset). We note that the selected HCP data were acquired with greater spatial and angular resolution, and higher *b* values compared with KU dataset. All HCP data were preprocessed by WU-Minn HCP and MGH-USC HCP Consortiums using methods that are described in Sotiropoulos et al. (2013) and Fan et al. (2014), respectively.

### Data analysis

#### Coregistration of functional and diffusion MR images to T1-weighted images

T1-weighted 3D anatomical images were aligned to the anterior commissure–posterior commissure (AC–PC) plane. To do this, the locations of AC and PC were manually defined in the T1-weighted images. These landmarks were then used to apply rigid-body transformation to align the anatomical images to the AC–PC plane.

Preprocessed fMRI and dMRI data were coregistered to the T1-weighted images in the AC-PC space, using a two-stage coarse-to-fine approach. This process enabled a direct spatial comparison between the tract endpoints and the ROIs within the same coordinates in each subject, in the later analyses.

#### Tractography

We used two complementary approaches to perform tractography. One is the ensemble tractography (Takemura et al. 2016a) based on the linear fascicle evaluation (LiFE; Pestilli et al. 2014; http://francopestilli.github.io/life/), which generates streamlines with various tractography parameters and removes those that do not contribute to predicting the diffusion signals. The advantage of this approach is that spurious streamlines that do not explain diffusion signals are not included in the resulting model. It also allows for evaluation of statistical significance of the estimated tract based on cross-validation (virtual lesion analysis; Pestilli et al. 2014; Takemura et al. 2016b; Leong et al. 2016). However, this approach requires a large amount of computational resources to optimise large-scale linear matrices composed of diffusion signals in every voxel acquired with multiple angular directions, as well as a mass of candidate streamlines (Takemura et al. 2016a). This makes it less practical to apply this framework to analysing data from large samples, until the ongoing work to reduce the necessary computational load of LiFE is completed (Caiafa and Pestilli 2017). Another limitation is that the current implementation of LiFE only accepts single-shell dMRI data (Pestilli et al. 2014).

The alternative approach is to exclude the ensemble tractography and LiFE from the analysis pipeline. At a cost of opting out statistical evaluation using the identical methods, we analysed the data of a large sample (i.e. 100 subjects) to assess the generality of our findings. Additionally, this alternative approach has a distinct advantage over the ensemble tractography and LiFE analyses, which is that it allows for tracking algorithms based on multi-shell dMRI data (Jeurissen et al. 2014). These two approaches can be complementary in validating the findings; one is designed to evaluate the statistical evidence on smaller samples, whereas the other simpler pipeline is well suited for assessing the generality across a larger number of subjects.

##### Ensemble tractography

For the six subjects of KU dataset (S1–S6) and four subjects from WU-Minn HCP dataset (S7–S10), we used the ensemble tractography (Takemura et al. 2016a) to estimate the white matter tract based on dMRI data from each subject. Measurements from the 2000 s/mm^2^ shell were extracted from the original WU-Minn HCP dataset and used for analyses.

Candidate streamlines were generated with MRtrix (Tournier et al. 2012), using CSD-based probabilistic tractography (step size 0.2 mm; maximum length 200 mm; minimum length 10 mm; fibre orientation distribution (FOD) amplitude stopping criterion 0.1). We used the entire grey matter–white matter interface region as a seed (Smith et al. 2012a), and the seed voxels were randomly selected from the mask for generating candidate streamlines. Tracking was terminated when a streamline reached outside the white matter mask. We used four different angular threshold settings (angular threshold 5.7°, 11.5°, 23.1°, 47.2°). Two million candidate streamlines were generated for each curvature parameter setting. We then selected those located within the white matter posterior to the most anterior optic-flow selective area (the cingulate sulcus visual area; CSv; Cardin and Smith 2010; Smith et al. 2012b, 2017) in each hemisphere, which were used in all subsequent analyses.

To obtain an optimised connectome model comprising streamlines generated with different curvature thresholds, we used the preselection method of the ensemble tractography (Takemura et al. 2016a). First, we separately optimised each connectome generated with a single curvature parameter using LiFE. We then selected 150,000 streamlines that contributed meaningfully to predicting the diffusion signals from each single-parameter connectome, and combined them to create a new candidate connectome (the ensemble tractography connectome; ETC; 600,000 candidate streamlines) in each hemisphere. Finally, LiFE was applied again to optimise the ETC. As a result, the optimised ETC included 103,664 streamlines on average per hemisphere. The optimised ETC has been shown to perform better than conventional connectome models, in terms of model accuracy and anatomical representation (Takemura et al. 2016a; https://github.com/brain-life/ensemble_tractography). Optimised ETCs were used for identification and statistical evaluation of SIPS.

##### Tractography analysis of a large sample

For dMRI data of the other 90 subjects (56 subjects from WU-Minn HCP and 34 subjects from MGH-USC HCP datasets), we performed probabilistic tractography based on multi-tissue CSD implemented in MRtrix (Jeurissen et al. 2014; step size 0.625 mm for WU-Minn HCP, 0.75 mm for MGH-USC HCP; angular threshold 45°; minimum length 2.5 mm for WU-Minn HCP, 3 mm for MGH-USC HCP; maximum length 250 mm; FOD amplitude stopping criterion 0.1). Measurements of all *b* values are used for estimating multi-tissue CSD. We used the entire grey matter–white matter interface region as a seed (Smith et al. 2012a), and the seed voxels were randomly selected from the mask for generating candidate streamlines. Tracking was terminated when a streamline reached the white matter mask. As a result, two million streamlines were generated. LiFE was not applied in this analysis.

#### Tract segmentation

We used the cortical ROIs defined by Freesurfer segmentation (Desikan-Killiany atlas; Desikan et al. 2006) to identify the white matter tract connecting the superior and inferior parts of the parietal cortex in the optimised connectome. For the main analysis, two cortical ROIs, one in the superior parietal cortex and the other in the inferior parietal cortex were created. The ROI in the superior part of the parietal cortex was defined as the combination of two Freesurfer ROIs: “precuneus” and “superior_parietal”. The combination of those two ROIs covered the positions of the functionally identified areas located in the superior parietal cortex (VIP, PcM, p2v). The Freesurfer ROI-labelled “supramarginal gyrus” was used as the ROI in the inferior parietal cortex, which covered the general region including PIC+.

The tract between the superior and inferior parts of the parietal cortex was identified as a group of streamlines having one of the endpoints near the superior parietal ROI and the other near the inferior parietal ROI (within 3 mm from each grey matter ROI; i.e. within two voxels from each grey matter ROI in KU, MGH-USC HCP datasets and three voxels in WU-Minn HCP dataset). Only streamlines terminating near the grey matter ROIs and not those passing through the grey matter ROIs were included, since tractography was anatomically constrained by the grey matter–white matter interface region used as a seed in generating candidate streamlines and did not allow for streamlines passing through the grey matter–white matter boundary to be generated (Smith et al. 2012a). The segmented tract was then refined by removing outlier streamlines. Those were streamlines that met the following criteria: (1) streamlines longer than the mean streamline length of the tract by ≥3 SD; (2) streamlines shorter than 15 mm; (3) streamlines of which positions are ≥3 SD away from the mean position of the tract (Yeatman et al. 2012b).

The MATLAB code used to identify SIPS in this study is available in Github repository [https://github.com/htakemur/SIPS] and also as part of the AFQ toolbox [Yeatman et al. 2012b; https://github.com/yeatmanlab/AFQ].

#### Estimating cortical endpoints of the tract

**Fig. 1:**
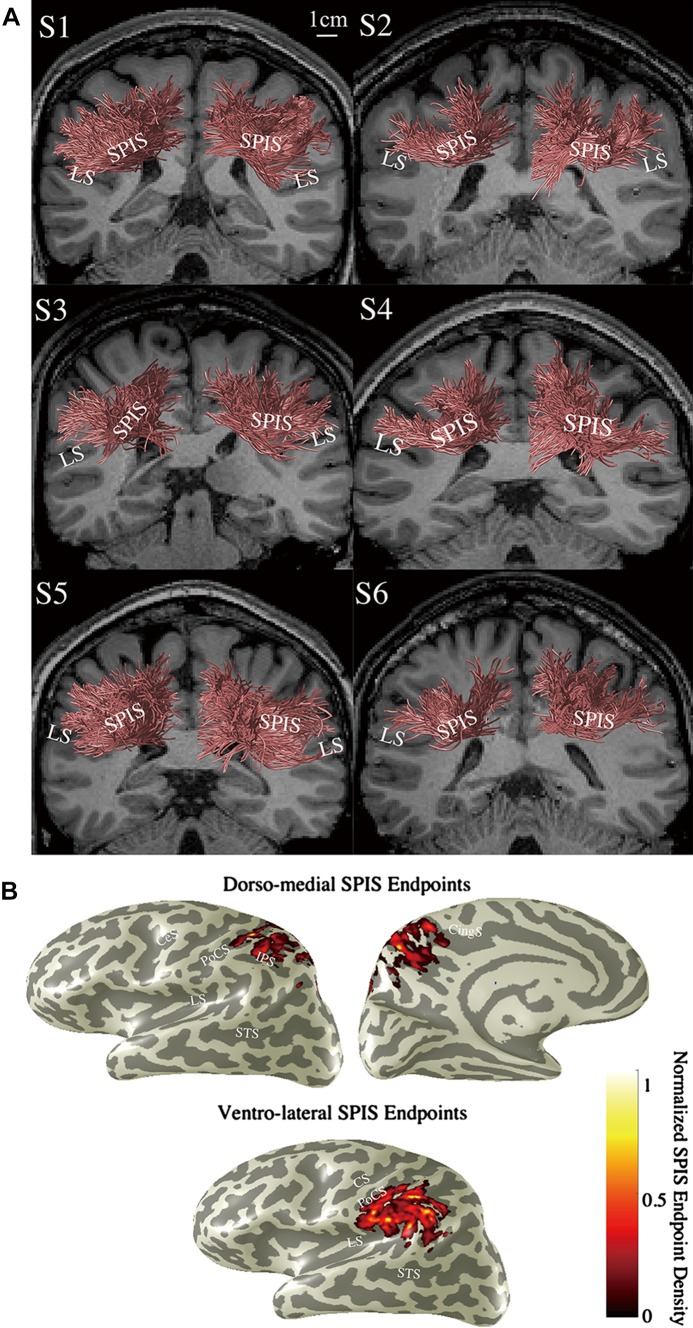
Human stratum proprium of interparietal sulcus (SIPS) estimated by tractography performed on dMRI data (KU dataset). **a** Coronal view of SIPS (*red*) in six subjects (S1–S6; KU dataset), identified by tractography (see “Materials and methods”). *Scale bar* (*white*) in the *S1 panel* indicates 10 mm. The background anatomical (T1-weighted) slice is located immediately anterior to the position of SIPS. *LS* lateral sulcus. **b** SIPS endpoints overlaid on the cortical surface (S1, left hemisphere). The spatial distance between SIPS endpoints and grey matter voxels was calculated to plot the number of SIPS streamlines having endpoints close to grey matter voxels (see “Materials and methods”). *CS* central sulcus, *PoCS* postcentral sulcus, *IPS* intraparietal sulcus, *STS* superior temporal sulcus, *CingS* cingulate sulcus

Streamlines terminate at the boundary between the white matter and grey matter. To estimate the SIPS endpoints in the cortical surface representation, the coordinates of the SIPS endpoints were collected, and the distance between those coordinates and the grey matter voxels was calculated. For each grey matter voxel, the number of the SIPS endpoints falling within a threshold distance (3 mm) was counted. The normalised endpoint counts are plotted on the inflated cortical surface in Fig. 1b. The same method was used in Takemura et al. (2016b).

#### Virtual lesion analysis

We conducted the virtual lesion analysis (Pestilli et al. 2014; Leong et al. 2016; Takemura et al. 2016b) on KU dataset to evaluate the statistical evidence supporting the existence of the white matter tract connecting the superior and inferior parts of the parietal cortex. For this analysis, we divided the dMRI data (KU dataset) into two sessions; one was used for performing tractography, and the other was used for computing cross-validated model prediction accuracy.

Two connectome models were used in the analysis; the optimised connectome and a lesioned connectome with the streamlines of interest (i.e. the streamlines that belong to the tract connecting the superior and inferior parts of the parietal cortex) removed. We computed prediction accuracy (root-mean-squared error; RMSE) of those models in predicting the diffusion signals. The set of dMRI data from the second session was used as the measured diffusion signals for cross-validation. Model accuracy is described as a ratio of RMSE (*R*_rmse_), and it represents prediction accuracy of each model with respect to test–retest reliability (for calculation methods, see Rokem et al. 2015; Takemura et al. 2016a). *R*_rmse_ = 1 indicates that the model accuracy for predicting the diffusion signals in the second dataset equals to test–retest reliability of the diffusion signals in the same voxel.

*R*_rmse_ was compared in all voxels touched by the lesioned streamlines (the streamlines that belong to SIPS). The complete set of streamlines that contribute to the prediction of the diffusion measurements in those voxels is called the path neighbourhood of SIPS (Wedeen et al. 2012). This path-neighbourhood includes SIPS itself and all of the other streamlines that pass through the voxels SIPS passes through. We calculated the distribution of *R*_rmse_ values in SIPS voxels with the entire path-neighbourhood included and removed SIPS, to figure out the weight of each of the remaining streamlines.

Finally, we compared the two *R*_rmse_ distributions using the strength of evidence, *S,* which is the difference in the mean *R*_rmse_ divided by the joint standard deviation (for technical detail; see Pestilli et al. 2014).

#### Spatial proximity between SIPS endpoints and functionally defined ROIs

To characterise the spatial proximity between the optic-flow selective ROIs and SIPS, we measured the proportion of grey matter voxels in each ROI (VIP, PcM, p2v and PIC+) located adjacent to the SIPS endpoints. We computed the three-dimensional distance between the endpoints of each SIPS streamline and each grey matter voxel included in the ROIs. We then calculated the proportion of voxels in each ROI located within a specific distance (thresholded at 3 or 4.5 mm in volumetric space) from any SIPS endpoints. This procedure is based on that reported in Takemura et al. (2016b).

We note that there are limitations to this analysis, due to the general difficulty in associating streamline endpoints and the grey matter surface (Reveley et al. 2015), particularly for dMRI data with a standard resolution such as those used in this study (i.e. KU dataset). The aim of this analysis was to examine the general spatial proximity between the tract endpoints and functionally defined ROIs, rather than to determine the projection pattern of the tract on the cortical surface.

#### Probabilistic atlas of SIPS

We created the probabilistic atlas of SIPS based on our 100-subject dataset following the method proposed in previous works (Bürgel et al. 2006; Catani and Thiebaut de Schotten 2012). The *b* = 0 images of each subject were normalised to the Montreal Neurological Institute 152 (MNI152) space using the MNI152 EPI template to obtain the affine transformation matrix. We then generated a binary visitation map of SIPS in each subject by assigning a value of 1 or 0 to each voxel depending on whether the voxel is intersected by any SIPS streamlines (Catani et al. 2007; Thiebaut de Schotten et al. 2008). This binary visitation map was normalised to MNI152 space according to the transformation matrix derived from the normalisation of the *b* = 0 images. Finally, we computed the percentage overlap by summing the normalised visitation maps of all subjects at each point in MNI152 space (Bürgel et al. 2006; Catani and Thiebaut de Schotten 2012). Supplementary Figures 2–5 show the visualisation of the percentage overlap with the overlap threshold at greater than 25% of the sample.

## Results

The primary aim of this study was to identify the white matter tract connecting the superior and inferior parts of the parietal cortex. By performing tractography on the dMRI data, we successfully identified SIPS in all subjects. We compared the tractography estimates with SIPS reported in the post-mortem fibre dissection studies (Sachs 1892; Vergani et al. 2014), and evaluated the statistical evidence for the estimates (Pestilli et al. 2014) as well as consistency across datasets. The tract was identified consistently across subjects and across datasets, and seems to correspond with the tract referred to as SIPS in previous fibre dissection studies (Sachs 1892; Vergani et al. 2014). Furthermore, we assessed the spatial relations between SIPS and the optic-flow selective sensory areas localised using fMRI.

### Anatomy of human SIPS

#### Tract trajectory and length

We analysed dMRI data of the six subjects from KU dataset using the ensemble tractography (Takemura et al. 2016a; see “Materials and methods”), whereby streamlines were generated using multiple parameter settings, and then optimised using LiFE (Pestilli et al. 2014; see “Materials and methods”). We identified a white matter tract having one set of endpoints near the superior parietal cortex and another near the inferior parietal cortex, based on the grey matter ROIs defined by Freesurfer (Desikan et al. 2006; Fischl 2012; see “Materials and methods”).

Figure 1a shows the group of streamlines that comprises the tract we identified in each hemisphere in six subjects from KU dataset, superimposed on a coronal slice of the T1-weighted anatomical image. The estimated tract, SIPS, primarily connects the superior and inferior parts of the parietal cortex and wraps around the intraparietal sulcus (IPS). In all subjects, SIPS in the two hemispheres are symmetrically oriented. The mean length of the estimated SIPS streamlines was 4.69 cm (SD 0.59, *N* = 12 hemispheres). Figure 1b shows the estimated SIPS endpoints on the cortical surface representation in one representative hemisphere (S1, left hemisphere). Most of the dorso-medial SIPS endpoints are not only in the parietal areas superior to the IPS but also in the medial areas such as the precuneus. The ventro-lateral endpoints of SIPS are in the supramarginal gyrus, parietal operculum and the posterior end of the lateral sulcus.

**Fig. 2:**
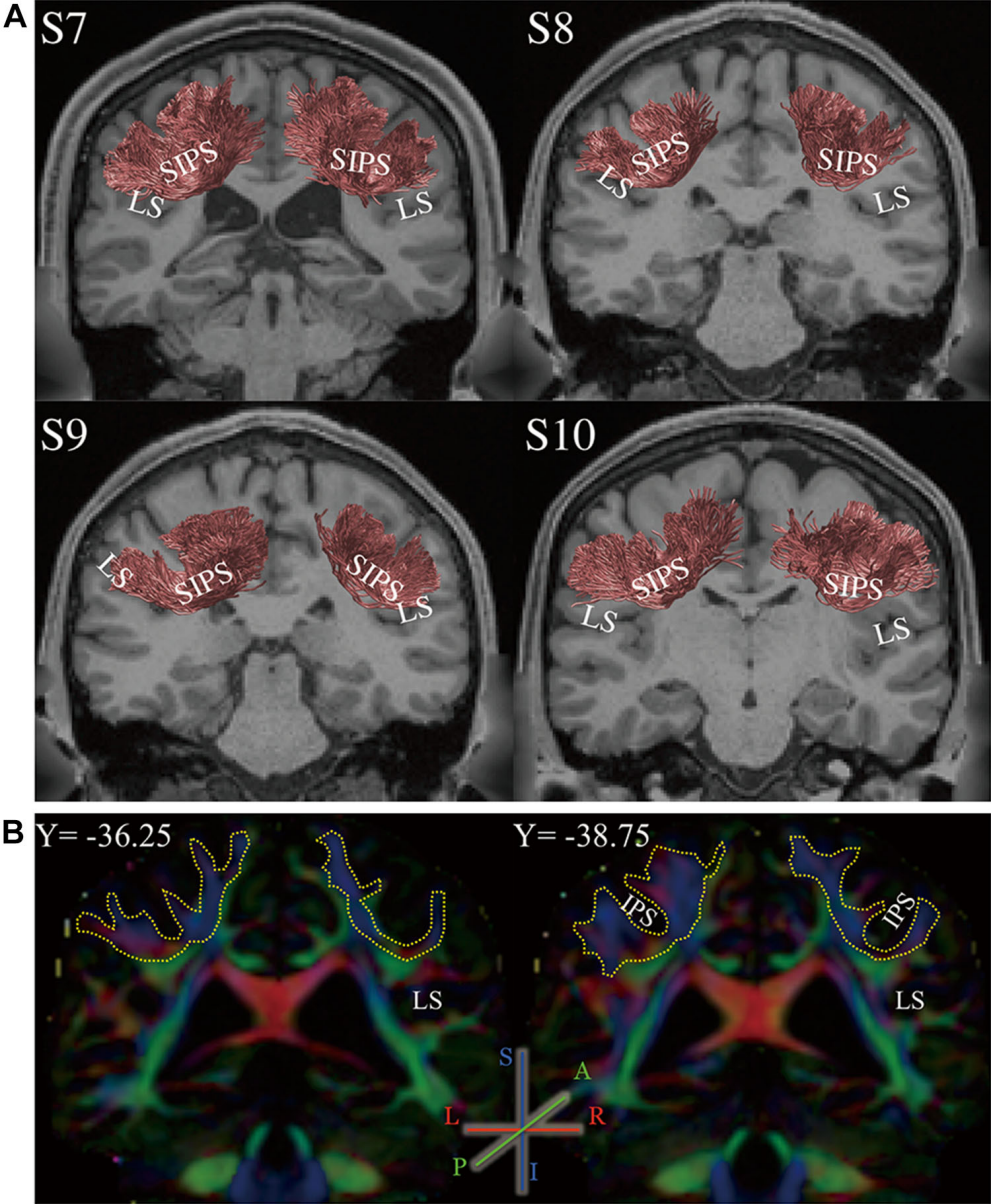
SIPS identified in HCP dataset. **a** Coronal view of SIPS (*red*) in four subjects (S7–S10) from HCP dataset, identified by tractography (see “Materials and methods”). The conventions are identical to those in Fig. 1a. **b** Position of SIPS highlighted in PDD map (S7, two representative coronal slices; the position of each slice is shown in ACPC coordinate). The *colour scheme* depicts the PDD in each voxel (*blue* superior–inferior; *green* anterior–posterior; *red* left–right). White matter portion connecting the dorso-medial and ventro-lateral regions wrapping around the intraparietal sulcus (IPS) that is predominantly *blue/purple* clearly illustrates the trajectory of SIPS. *Yellow*-*dotted line* highlights the position of SIPS

Additionally, we analysed the publicly available HCP data (WU-Minn HCP dataset; Van Essen et al. 2013) to assess the consistency of SIPS results across datasets. Figure 2a demonstrates SIPS in four subjects from WU-Minn HCP dataset, identified using the identical methods to those used for KU dataset. The estimated position and trajectory of SIPS are consistent with those in KU dataset (Fig. 1a).

Figure 2b shows a principal diffusion direction (PDD) map illustrating the position of SIPS in one representative WU-Minn HCP subject (S7). PDD is often used to identify the major white matter tracts, and it allows for tract identification independent of the selection of tractography methods (Pajevic and Pierpaoli 1999; Wakana et al. 2004; Yeatman et al. 2013; Takemura et al. 2016b). This PDD map based on WU-Minn HCP dataset clearly shows the existence of a tract travelling between the medial side of superior parietal cortex, and lateral inferior regions around parietal operculum and posterior part of lateral sulcus.

To assess the generality of our findings, we analysed dMRI data of 90 subjects from WU-Minn HCP and MGH-USC HCP datasets using a simpler analysis pipeline (see “Materials and methods”). Supplementary Figure 1 demonstrates that SIPS was also consistently observed in those subjects. We also describe the population average of the position of SIPS in MNI152 space in Supplementary Figures 2–5.

The results of tractography, which is consistent across a large number of subjects and three independent datasets, as well as voxelwise evidence of SIPS that is not based on tractography, further corroborate the evidence for SIPS. Furthermore, SIPS described here is consistent with a short parietal association bundle reported in a series of white matter atlas works (Oishi et al. 2008, 2011; Zhang et al. 2010; Guevara et al. 2017). However, the atlas does not provide additional information regarding the provenance of SIPS, making our study the first to report the detailed anatomical characteristics of this tract using in vivo dMRI methods and to compare SIPS identified based on in vivo dMRI data to post-mortem findings, as described below.

#### Comparison with fibre dissection studies

We compared the anatomical position and shape of SIPS identified from dMRI data with post-mortem fibre dissection studies. SIPS has been documented in two previous post-mortem fibre dissection studies; in the classical work by a German neurologist Heinrich Sachs (1892) and more recently by Vergani et al. (2014). Sachs referred to this tract as stratum proprium fissurae interparietalis in his report, which was later rephrased by Vergani et al. (2014) as stratum proprium of interparietal sulcus (SIPS). We adopt this term, SIPS, to refer to the tract estimated in the present study.

**Fig. 3:**
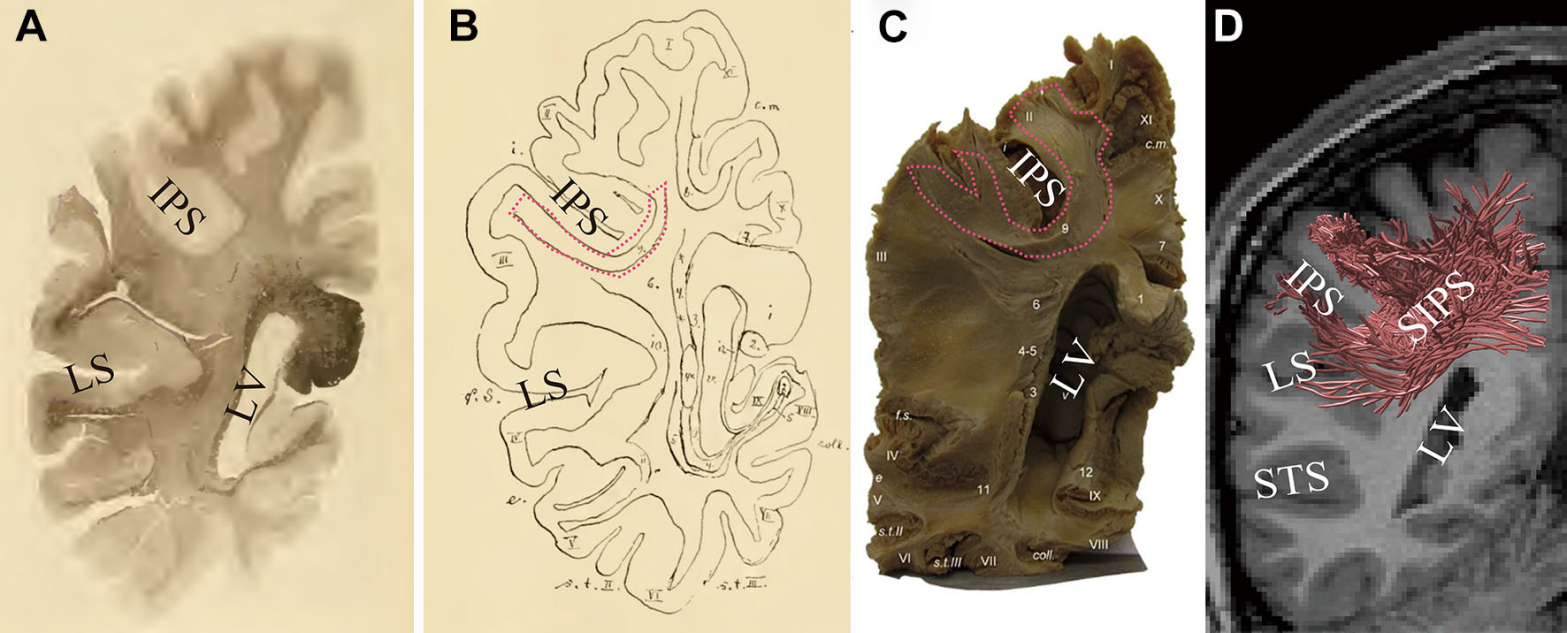
Tractography results are consistent with classical and modern fibre dissection studies. **a** White matter tract referred to as SIPS on a coronal histological slice and **b** SIPS (*red outline*) in the schematic diagram of the fibres visualised in the photo in **a** (Sachs 1892). Sachs (1892) noted that this slice is approximately 75 mm anterior to the occipital pole (Forkel et al. 2015). **c** SIPS identified in a post-mortem human brain in the modern fibre dissection study (right hemisphere; Vergani et al. 2014). The position of SIPS is highlighted with *red outline*. Reproduced from Vergani et al. (2014) with permission. **d** SIPS estimated by tractography (*red lines*) in one representative hemisphere (S1; right hemisphere). The image has been flipped (for the original image, see Fig. 1a) so that the slice corresponds with the fibre dissection studies. The background coronal slice (ACPC coordinate, *Y* = −44; approximately 65 mm anterior to the occipital pole) is located immediately anterior to the estimated SIPS. The position and the trajectory of SIPS are qualitatively consistent with those of SIPS reported in the fibre dissection studies (**a**–**c**). *LV* lateral ventricle, *STS* superior temporal suclus, *IPS* intraparietal sulcus

Figure 3 compares the position of SIPS in Sachs’s study (1892; Fig. 3a, b) and in Vergani’s study (2014; Fig. 3c), with the tract we identified using tractography (Fig. 3d). SIPS identified in this study is consistent with SIPS reported in the fibre dissection studies in several aspects. In terms of its spatial relations with cortical landmarks, SIPS wraps around the intraparietal sulcus, and connects the parietal cortex and the dorsal bank of the lateral sulcus. The position of SIPS on the coronal slice is also consistent. The coronal slice of the anatomical image onto which SIPS estimated by tractography is superimposed in Fig. 3d was chosen carefully so that it corresponded with the slices used in the fibre dissection studies as closely as possible. Whilst it is not possible to perfectly match the position of the slice between our MRI data and fibre dissection studies, it is qualitatively consistent across the four presentations in Fig. 3, in terms of the positions of the sulci (i.e. the lateral sulcus and the intraparietal sulcus) and the lateral ventricle. Although SIPS in the three studies cannot be compared quantitatively due to the difference in the methodology used, Fig. 3 highlights that the position and trajectory of SIPS identified with our dMRI data agree with those of SIPS reported in the fibre dissection studies.

#### Position of SIPS with respect to major white matter tracts

**Fig. 4:**
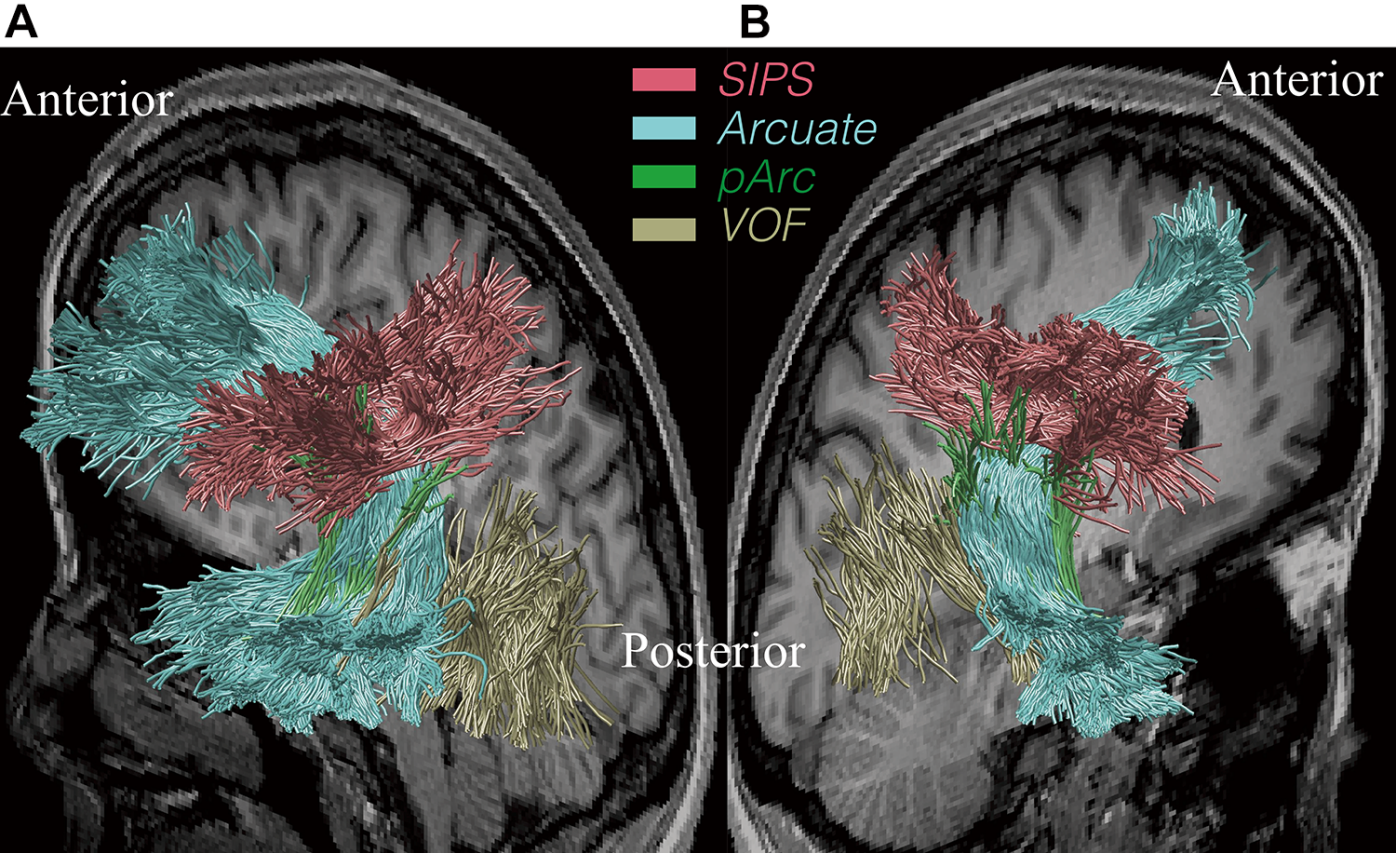
Position of SIPS with respect to other tracts**. a** Position of SIPS with respect to other tracts in the left hemisphere of one representative subject (S1). The background T1-weighted image is a sagittal slice in the medial portion of the brain. SIPS (*red*) is located superior to the vertical occipital fasciculus (VOF; *yellow*) and posterior arcuate (pArc; *green*). SIPS lies on the superior surface of, and crosses with the arcuate fasciculus (arcuate; *light blue*). **b** Position of SIPS with respect to other tracts in the right hemisphere in the same subject (S1)

Figure 4 shows SIPS overlaid on a sagittal plane of the T1-weighted image, along with other major white matter tracts reported in previous studies; the arcuate fasciculus (AF; Catani et al. 2002; Wakana et al. 2004), posterior arcuate (pArc; Catani et al. 2005; Weiner et al. 2016) and vertical occipital fasciculus (Yeatman et al. 2013, 2014b; Duan et al. 2015; Takemura et al. 2016b) in one representative subject (S1).

SIPS is located adjacent to AF, and in fact, SIPS intersects with the dorsal surface of AF. This crossing may be one of the reasons that this tract has been relatively neglected in the literature, as resolving crossing fibres is one of the critical limitations of the diffusion tensor-based approach (Frank 2001; Tournier et al. 2012). Interestingly, the intersection between SIPS and AF may explain the pattern of previous dMRI results along AF. Yeatman et al. (2011) investigated the fractional anisotropy (FA; Basser and Pierpaoli 1996) along AF, and found that there is a large dip in the FA value along the length of the fasciculus in the vicinity of the temporal cortex. Yeatman et al. (2011) suggest that this dip is not only partially accounted for by the sharp curvature of AF but also by partially voluming with crossing fibres. Since the location of this dip along the trajectory of AF coincides with the position of SIPS, it seems plausible that this is where AF intersects with SIPS.

SIPS is also located near pArc, but the trajectory and endpoints of SIPS are distinct from pArc, which connects the parietal cortex and the anterior inferior temporal cortex.

Although there are a few neighbouring tracts, some of which cross or kiss SIPS, the differences in the trajectory and locations of endpoints between those known tracts and SIPS clarify that SIPS is a distinct tract.

### Statistical evidence in support of SIPS

To evaluate the strength of statistical evidence supporting the existence of SIPS, we used the virtual lesion methods (Honey and Sporns 2008; Pestilli et al. 2014; Leong et al. 2016; Takemura et al. 2016b). We first computed the cross-validated prediction accuracy for diffusion signals (ratio of RMSE; *R*_rmse_; Rokem et al. 2015; Takemura et al. 2016a) in models with lesioned and unlesioned SIPS. We then compared the distribution of *R*_rmse_ of the two models to predict the diffusion signals within SIPS voxels (see “Materials and methods” for details). We quantify the strength of evidence (*S*) in support of the SIPS by calculating the difference of *R*_rmse_ in lesioned and unlesioned models divided by the standard deviation of the *R*_rmse_ (Pestilli et al. 2014).

**Fig. 5:**
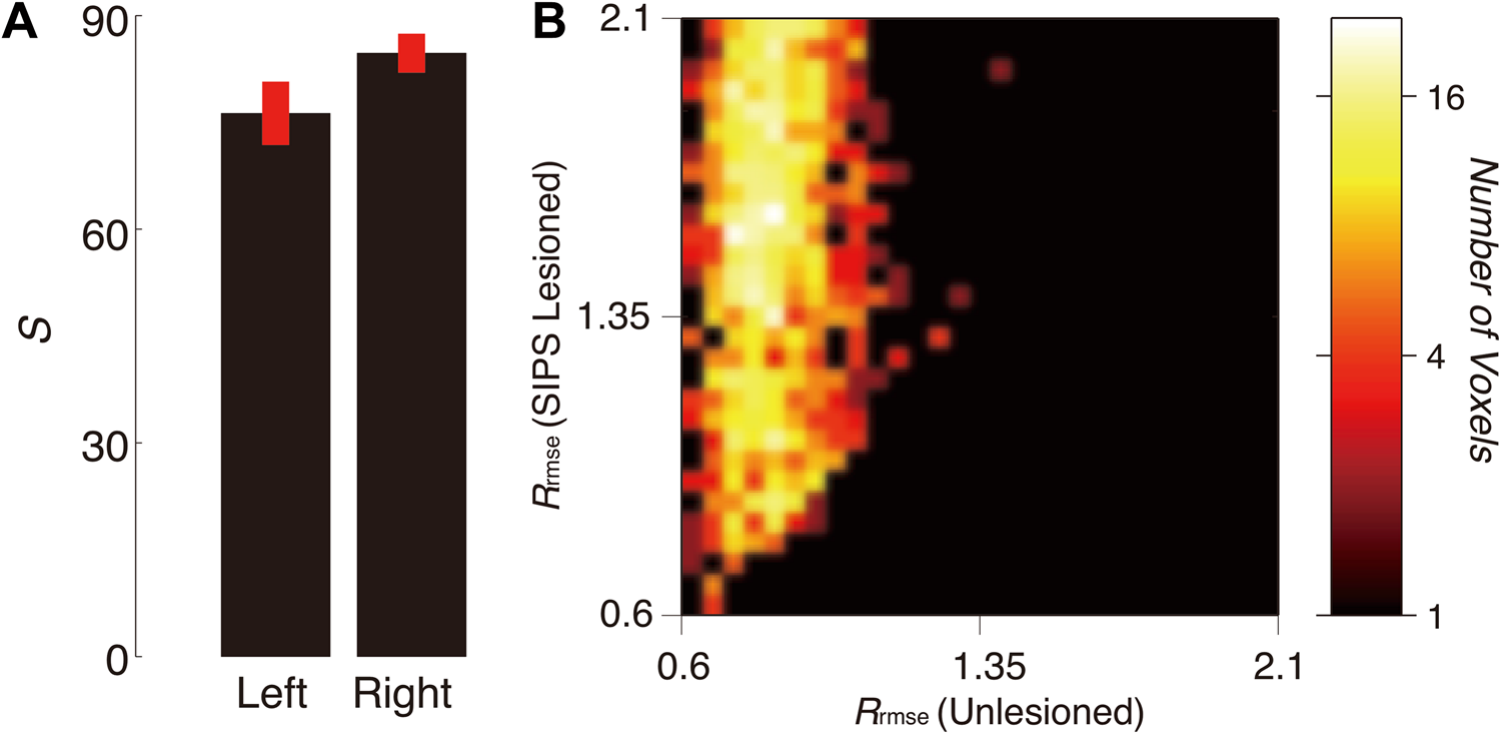
Statistical evidence in support of SIPS**. a** Mean *S* in support of left and right SIPS across subjects. *Error bars* depict ±1 SEM across subjects. **b** Two-dimensional histogram comparing the model accuracy (*R*_rmse_) between the lesioned and unlesioned models (*horizontal axis* unlesioned model; *vertical axis* lesioned model) for SIPS in the left hemisphere in one representative subject (S1). Prediction accuracy is substantially lower with the lesioned model. *Colour bar* (*right panel*) indicates the number of voxels

Figure 5a describes the mean and variance of the statistical evidence for SIPS across subjects, yielded by the virtual lesion analysis. The mean strength of statistical evidence for SIPS was *S* = 76.25 (SD 10.87) for the left hemisphere, and *S* = 84.7 (SD 6.72) for the right hemisphere. Figure 5b describes the two-dimensional histogram of *R*_rmse_ in the SIPS lesioned and unlesioned models for the left hemisphere in one representative subject (S1). In many voxels, the SIPS-lesioned model showed substantially lower model accuracy (higher *R*_rmse_) as compared with the unlesioned model, indicating that SIPS is necessary to explain the diffusion signals within those voxels. Thus, in addition to the results of tractography and their consistency with the findings of previous post-mortem studies at visual inspection, there is strong statistical evidence supporting the existence of SIPS.

### SIPS and its relations with optic-flow selective cortical areas

With the subjects in KU dataset, we further conducted fMRI experiments to localise cortical sensory areas selective for optic-flow stimulation to examine the spatial proximity between the SIPS endpoints and those functionally defined areas.

#### Functional localisation

**Fig. 6:**
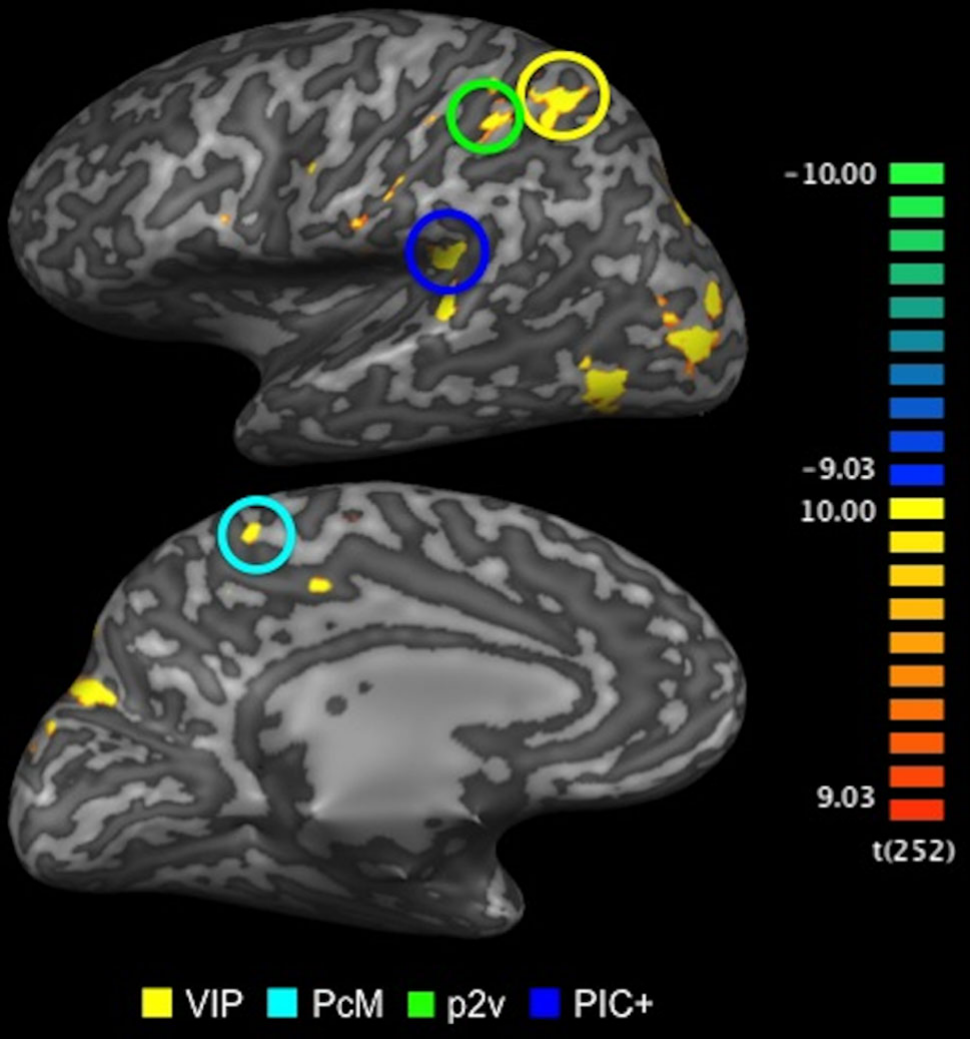
Optic-flow selective areas localised using fMRI. Cortical areas that showed significantly greater BOLD responses to optic-flow stimulus than to random-motion stimulus (*p* < 0.005, uncorrected). Activation maps are superimposed on the inflated cortical surface of the left hemisphere in one representative subject (S1). *Colour-coded bar* (*right panel*) indicates statistical *t* values (degree of freedom indicated in *brackets*). Four of the cortical areas selective for optic flow (VIP, PcM, p2v and PIC+) were successfully identified

To localise the cortical areas selective for optic-flow stimulation, blood-oxygen-level-dependent (BOLD) responses to the coherent optic-flow stimulus was contrasted against those to the random-motion stimulus. We identified four of the cortical areas known to be selective for optic flow (Fig. 6; VIP, p2v, PcM, PIC+; Cardin and Smith 2011; Uesaki and Ashida 2015). Areas VIP, p2V and PcM are located in the superior part of the parietal cortex, and PIC+ in the posterior end of the lateral sulcus. The locations of those areas in Talairach coordinates were consistent with those of the corresponding areas reported in previous studies (Cardin and Smith 2011; Frank et al. 2014; Uesaki and Ashida 2015). All four areas were successfully identified in nine hemispheres. In the other three hemispheres, either PcM or VIP was not identified.

#### SIPS endpoints and optic-flow selective cortical areas

Subsequently, we examined the spatial proximity between the cortical areas selective for optic flow (Fig. 6) and the SIPS endpoints. Although there is a limitation to use tractography for identifying the tract endpoints in the grey matter (Reveley et al. 2015), it is still useful to understand how closely functionally defined ROIs are located to the tract endpoints to infer any potential implication of the tract in information transmission during optic-flow processing. We analysed the general spatial proximity between the SIPS endpoints and the optic-flow selective areas (VIP, PcM, p2v and PIC+).

**Fig. 7:**
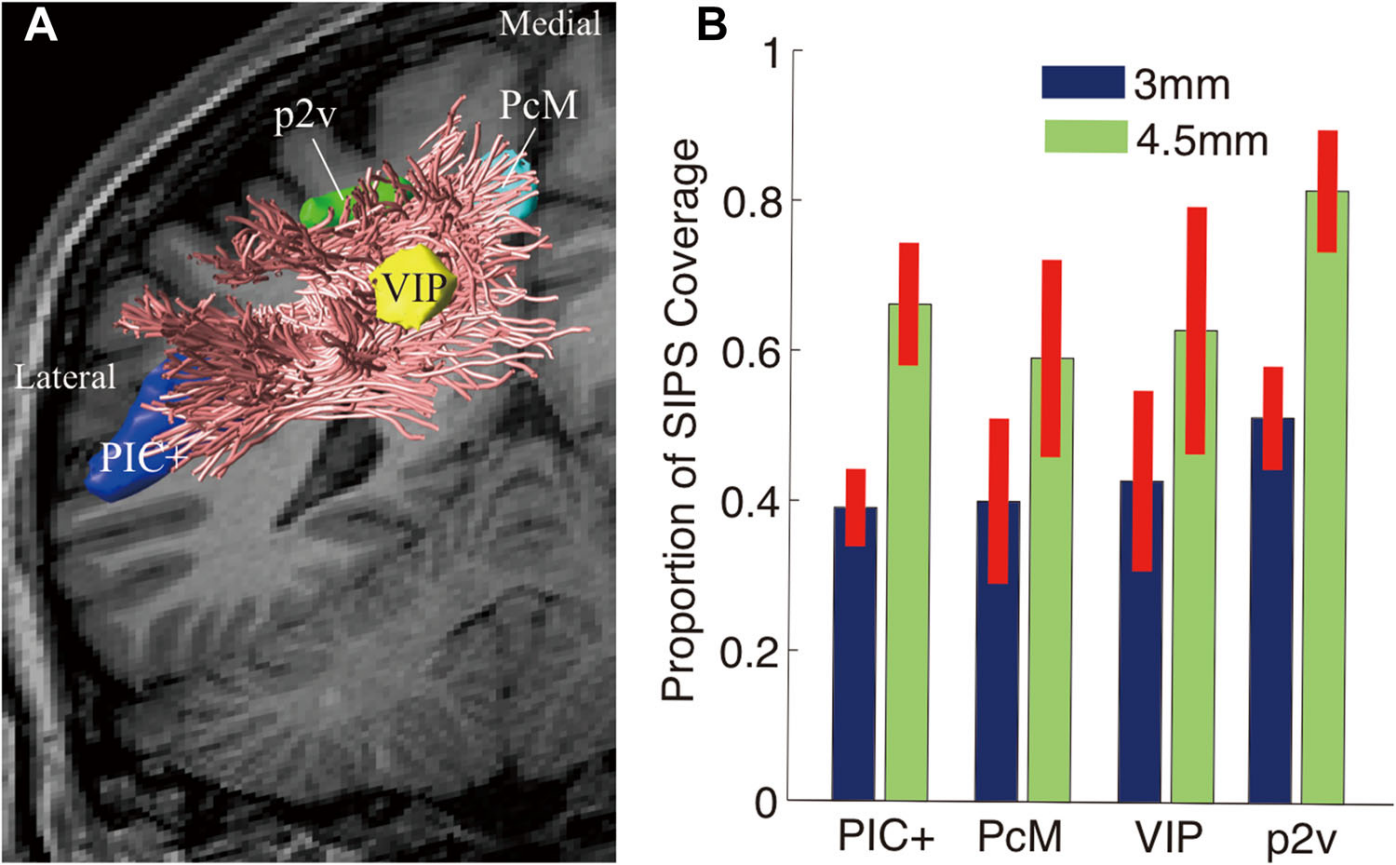
Spatial proximity between SIPS endpoints and cortical ROIs selective for optic-flow stimulation. **a** Position of SIPS (*magenta lines*) in relation to the cortical ROIs identified by optic-flow stimulation using fMRI (*dark blue* PIC+; *light blue* PcM; *yellow* VIP; *green* p2v) in the left hemisphere in one representative subject (S1). **b** SIPS map coverage across all hemispheres. *Vertical axis* represents the proportion of voxels in each ROI within 3 mm (*blue*) and 4.5 mm (*green*) from SIPS endpoints. *Error bars* indicate ±1 SEM across hemispheres

Figure 7a depicts the relative position of SIPS with respect to the cortical areas selective for optic flow, in the left hemisphere of one representative subject (S1; see Supplementary Figure 6 for other examples). PIC+ is located in the posterior end of the lateral sulcus, near the ventro-lateral endpoints of SIPS. Three of the optic-flow selective areas (VIP, p2v and PcM) in the parietal cortex are located near the dorso-medial endpoints of SIPS. Whilst the ventro-lateral endpoints are observed in the vicinity of PIC+ in a consistent manner across hemispheres, there are some degrees of variability in the spatial proximity between the superior parietal ROIs and dorso-medial endpoints of SIPS across hemispheres (Supplementary Figure 6). This variability may be due to the limitation of tractography in identifying the exact tract endpoints near smaller cortical regions located in the gyrus walls (Reveley et al. 2015). Figure 7b summarises the proportion of grey matter voxels in each optic-flow selective area located near the SIPS endpoints (see “Materials and methods”). Approximately 40 and 80% of voxels in each grey matter ROI are located in the vicinity of the SIPS endpoints, depending on the distance threshold for defining the spatial proximity between the grey matter voxels and tract endpoints (i.e. proximity was thresholded at 3 or 4.5 mm). It seems highly likely that SIPS is part of the anatomical connection between the optic-flow selective areas in the superior parietal cortex (VIP, p2v and PcM) and the PIC+.

## Discussion

Stratum proprium of interparietal sulcus (SIPS) was originally discovered in a post-mortem fibre dissection study by Sachs (1892) and was reproduced in another post-mortem study by Vergani et al. (2014). Here, SIPS was successfully identified in the living human brain, using dMRI and tractography.

### Comparison of SIPS results with anatomical studies

In this study, we investigated a white matter tract that has been largely overlooked in the visual and cognitive neuroscience, SIPS, using dMRI-based tractography and fascicle evaluation techniques. In spite of the challenges of using tractography to study little-investigated white matter tracts, SIPS was consistently identified across subjects and datasets. Between our dMRI results and the findings of the following anatomical studies, there is converging evidence supporting the existence of SIPS.

#### Human post-mortem fibre dissection studies

Most importantly, our results are consistent with human post-mortem fibre dissection studies (Fig. 3; Sachs 1892; Vergani et al. 2014). In those studies, SIPS was found to be located immediately posterior to the central sulcus, wrapping around the intraparietal sulcus, and to range between the superior parietal cortex and the lateral fissure. The position and the trajectory of SIPS reported in the post-mortem fibre dissection studies are consistent with those of SIPS identified in vivo in three independent dMRI datasets in this study (Figs. 1, 2; Supplementary Figures 1–5).

To our knowledge, the first description of SIPS appeared in the atlas by Heinrich Sachs (1892); a German neurologist and neuroanatomist who studied under Wernicke. Sachs’s atlas (1892) describes the white matter tracts in the post-mortem human brain in great detail, including the U-fibre system which has not been studied extensively in the living human brain. One of the short association tracts described is a tract termed stratum proprium fissurae interparietalis. Despite its relevance to perceptual and cognitive neuroscience, Sachs’s atlas has been largely overlooked in the literature partly due to the lack of translation of the atlas from German to English (see Forkel et al. 2015; for a historical review and English translation of the atlas). SIPS documented in this classical atlas was recently reproduced in a modern fibre dissection study by Vergani et al. (2014).

Our results describe the characteristics of SIPS identified in the living human brain, using modern neuroimaging techniques, which are highly consistent with the findings of the human post-mortem studies; hence providing further evidence for SIPS.

#### Macaque tracer study

**Fig. 8:**
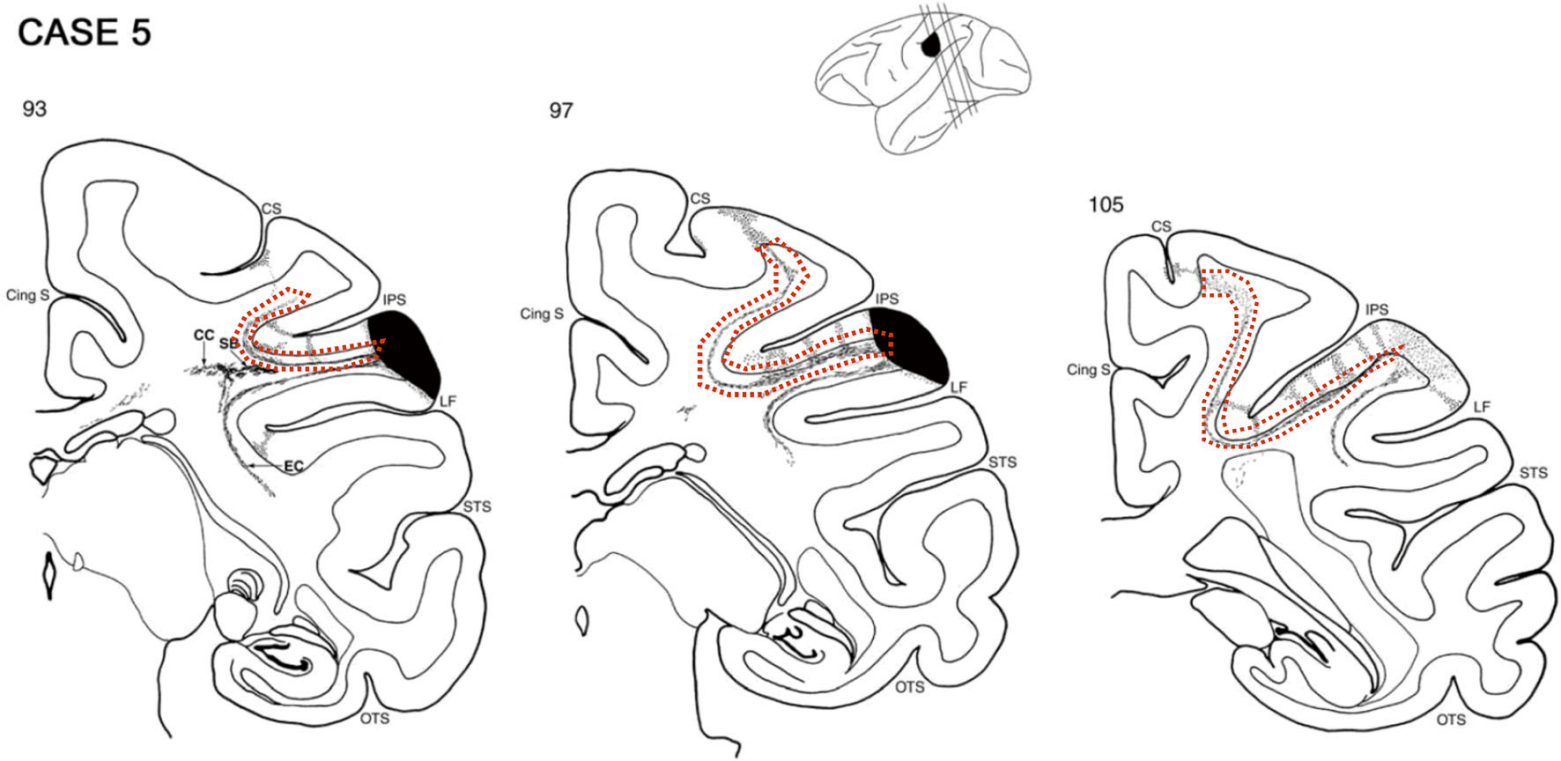
A fibre bundle wrapping around the intraparietal sulcus reported in a macaque tracer study. This figure is reproduced from page 124 of Schmahmann and Pandya (2006, by permission of Oxford University Press, USA; http://www.oup.com). *Each panel* represents a coronal slice in the macaque brain (*left panel* anterior slice; *right panel* posterior slice). *Cortical area marked in black* is the injection site of anterograde tracer. *Areas highlighted with dotted red lines* in the white and grey matters indicate the axonal connections from the injection site, which defines a white matter tract wrapping around the intraparietal sulcus

Additionally, we note that a tract similar to human SIPS in the macaque brain has been reported in a tracer study. In their extensive study, Schmahmann and Pandya (2006) injected retrograde tracers into the macaque brain, and inspected the trajectory of white matter tracts from the injection sites. They reported several major white matter tracts seemingly homologous to human major white matter tracts identified in dissection studies (such as the inferior longitudinal fasciculus, and the superior longitudinal fasciculus); and those findings were later substantiated by macaque dMRI results (Schmahmann et al. 2007). In addition to the major white matter tracts, Schmahmann and Pandya (2006) also reported a fibre bundle wrapping around the intraparietal sulcus (Fig. 8). They note (page 120):

A dorsal fibre bundle lies subjacent to the cortex of the lower bank of the IPS and terminates in a columnar manner in area POa and in area IPd (Scs. 105, 113). These fibres continue medially and then curve around the depth of the IPS to ascend in the white matter of the superior parietal lobule. They terminate in area I in a columnar manner and then first layers of area 3b and 3a in the caudal bank and depth of the central sulcus (Sc. 105). Further caudally, these medially directed fibres terminate in a columnar manner in area 2 (Sc. 113).

Because of the compelling similarity between this fibre bundle identified in the macaque brain and human SIPS in terms of their anatomical positions and shapes, it could be hypothesised that this fibre bundle in macaque may be the homologue of human SIPS identified in this study.

Whilst the white matter structure of the macaque brain may be different from that of the human brain to some extent (Rilling et al. 2008), the fact that there is a white matter tract in the macaque brain that largely resembles human SIPS is encouraging for future investigations on human–macaque homology with respect to SIPS. There is a growing trend in neuroanatomy to use dMRI methods to compare the macro-scale white matter anatomy of the human brain and that of the macaque brain (Schmahmann et al. 2007; Oishi et al. 2011; Thiebaut de Schotten et al. 2011; Jbabdi et al. 2013; Mars et al. 2016; Takemura et al. 2017), which complements studies that investigate human–macaque homology of cortical maps using fMRI (Brewer et al. 2002; Tsao et al. 2003, 2008; Wade et al. 2008; Goda et al. 2014; Kolster et al. 2014). It will be beneficial to study the precise anatomy of SIPS both in humans and macaques, to integrate the insights from macaque electrophysiology as well as tracer studies (Thiebaut de Schotten et al. 2012), and human fMRI studies investigating the neuronal network for multisensory integration guiding self-motion perception.

### Functional localisation of optic-flow selective sensory regions

Recent fMRI studies have shown that the sensory areas in the superior parietal regions (VIP, PcM, p2v) as well as an area around the posterior end of the lateral sulcus and parietal operculum (PIC+) are activated by optic-flow stimulation (Wall and Smith 2008; Cardin and Smith 2011; Greenlee et al. 2016). As in Uesaki and Ashida (2015), this study employed the functional localiser based on that described in Pitzalis et al. (2010), to identify VIP, PcM, p2v and PIC+. The locations of those regions are consistent with those of the counterparts reported in previous studies (Cardin and Smith 2011; Uesaki and Ashida 2015).

We note that the definition and terminology of the area referred as PIC+ in this study have been debated in the literature. In some earlier publications (Wall and Smith 2008; Cardin and Smith 2010, 2011; Uesaki and Ashida 2015), an area identified using optic-flow localisers was referred to as the parieto-insular vestibular cortex (PIVC) and was considered to be involved in integrating visual and vestibular information to guide self-motion perception. However, a recent vestibular fMRI study showed that PIVC is selectively responsive to vestibular stimulation, and is unlikely to be activated by visual stimulation (Frank et al. 2016; Greenlee et al. 2016). Frank et al. (2014) also suggested that PIVC and the area activated by visual stimulation, which is referred to as “PIC” in their study, are two independent areas. Their findings show that PIVC is purely vestibular, whilst PIC is predominantly visual but also processes vestibular information. Here, we use “PIC+” to refer to the area around the posterior end of the lateral sulcus and parietal operculum, activated during optic-flow stimulation, as we did not examine the responsiveness of the area to vestibular stimuli.

Results suggest that the ventro-lateral endpoints of SIPS are near PIC+ (Fig. 7), but it is unclear whether these endpoints are also located near PIVC identified in vestibular fMRI studies (Frank and Greenlee 2014; Frank et al. 2014; Greenlee et al. 2016). Considering the proximity between PIC and PIVC (Frank and Greenlee 2014; Frank et al. 2014; Greenlee et al. 2016), it is possible that the ventro-lateral endpoints of SIPS are also adjacent to PIVC. Future studies should assess whether PIVC is directly connected to the superior part of the parietal cortex through SIPS, or indirectly connected via short-range connections with PIC+, to construct a more comprehensive model to understand how visual and vestibular signals are transmitted between these areas to guide self-motion perception.

### SIPS and its implication in multisensory integration

Optic flow is a moving pattern on the retina caused by the relative motion between the observer and the scene, and is one of the most important visual cues to the estimation of self-motion (Gibson 1950, 1954; Warren and Hannon 1988). However, in most cases, perception of self-motion depends on integration of optic-flow information and signals from other sensory modalities such as the vestibular system. To understand the neuronal mechanism involved in the estimation of self-motion, it is important to elucidate how the visual and vestibular signals are integrated when we observe optic flow. Previous fMRI studies investigating the cortical areas selective for optic-flow and vestibular stimuli suggest that the sensory areas in the parietal cortex are involved in visuo-vestibular integration necessary for self-motion estimation (Wall and Smith 2008; Cardin and Smith 2011; Greenlee et al. 2016). Yet, the white matter anatomy that supports the communication amongst those areas has received very little attention in the literature of visual and cognitive neuroscience, even though the existence of SIPS has been known for over a century (Sachs 1892; Vergani et al. 2014).

One of the biggest advantages of the dMRI-based approach is that the positions of estimated white matter tracts and functionally localised cortical areas can be compared in the brain of the same individual. This is particularly important to hypothesise the types of information that are transferred via the tracts of interest (Kim et al. 2006; Greenberg et al. 2012; Yeatman et al. 2013; Takemura et al. 2016b; Rokem et al. 2017). We combined dMRI and fMRI, and analysed the spatial proximity between the SIPS endpoints and the optic-flow selective cortical areas localised within the same subjects. Results show that the dorso-medial SIPS endpoints are near VIP, PcM and p2v, and the ventro-lateral SIPS endpoints near PIC+, despite some variability in the spatial proximity between the superior parietal ROIs and dorso-medial endpoints of SIPS across hemispheres (Fig. 7; Supplementary Figure 6). These cortical areas have been associated with the convergence of visual and vestibular information regarding self-motion (Fetsch et al. 2009; Prsa et al. 2012; Uesaki and Ashida 2015; Kleinschmidt et al. 2002; Kovács et al. 2008; Wiest et al. 2004; Butler et al. 2010). Our results and those findings together suggest that communication between VIP, PcM, p2v and PIC+ likely plays a crucial role in multisensory integration necessary for accurate perception of self-motion, and that it is supported by SIPS. The spatial relationship between SIPS and the optic-flow selective areas will have implications for interpreting the consequences of white matter lesions that include SIPS, or exploring the neuronal basis of individual differences in self-motion perception.

### Limitations and directions for future research

Our findings show that SIPS is an important structure supporting communication amongst sensory areas in the parietal cortex. It must be noted, however, that it is possible that our results represent only a subset of SIPS. Our tractography results are based on in vivo dMRI data with 1.25–2 mm isotropic spatial resolution, but LiFE analysis generally supports larger portions of fibre tracts as the data resolution improves (Pestilli et al. 2014; Takemura et al. 2017). Tractography based on data with higher resolutions would likely allow for the extraction of a larger portion of SIPS. Likewise, estimation of cortical endpoints would be more accurate with data of better quality, as some cortical endpoints are still missed even with the best dMRI data currently attainable (Reveley et al. 2015).

Other limitations that should be considered include the lower *b* value (1000 s/mm^2^) used in the acquisition of KU dataset. It has been suggested that lower *b* values are not optimal for resolving crossing fibres (Tournier et al. 2004; Alexander and Barker 2005), despite their relatively higher signal-to-noise ratio. To compensate for this limitation, we included HCP data acquired with higher *b* values, higher spatial and angular resolution from a large number of subjects. Results demonstrated the compelling consistency in the tractography results across the three datasets. This approach complements the relative disadvantage of the current version of LiFE that it only accepts single-shell data. Contrary to the single-shell approach, multi-shell approaches can be used to generate alternative matrices, which provide additional information regarding tissue microstructures that cannot be captured at a voxel level using single-shell approaches.

It should also be noted that, although SIPS is discussed mainly within the contexts of multisensory integration and optic-flow processing in this article, the SIPS endpoints appear to be near the cortical areas involved in other cognitive functions such as attention (Corbetta and Shulman 2002; Yantis et al. 2002; Bisley and Goldberg 2003), memory (Cabesa et al. 2008; Koenigs et al. 2009; Uncapher and Wagner 2009; Chun and Johnson 2011), motor sequence learning (Rizzolatti and Luppino 2001), visuomotor control (Culham et al. 2006), decision making (Platt and Glimcher 1999), body-ownership (Blanke 2012) and social cognition (Decety and Lamm 2007). To further understand the implications of SIPS in relation to human behaviour, it may be useful to assess the relationship between individual differences in diffusion properties along SIPS (e.g. FA) and behavioural measures, as has been done for other white matter tracts in previous studies (Genç et al. 2011; Yeatman et al. 2012a; Tavor et al. 2014; Gomez et al. 2015; Leong et al. 2016).

On the other hand, for relatively small tracts like SIPS, especially if they cross or kiss other major tracts like AF (Fig. 3), it is difficult to interpret the results regarding diffusion properties derived from diffusion tensor-based analyses. Possible solutions to these limitations include acquiring dMRI data at a higher angular resolution with multiple *b* values, use of other dMRI techniques (e.g. neurite orientation dispersion and density imaging; NODDI; Zhang et al. 2012), and combining dMRI data with quantitative MRI measurements, to evaluate the physical properties of SIPS in a manner relatively independent from fibre crossings (Dell’Acqua et al. 2013; Mezer et al. 2013; Yeatman et al. 2014b; Mohammadi et al. 2015; Stikov et al. 2015; Weiskopf et al. 2015). Future dMRI studies should examine the properties of SIPS in relation to cognitive functions, as well as development and diseases, with consideration to shortcomings of currently available MRI techniques. To facilitate further research on SIPS, we describe the methods and provide open-source implementations [https://github.com/htakemur/SIPS; https://github.com/yeatmanlab/AFQ].

## Conclusion

This study identified the white matter tract, SIPS, in the living human brain using dMRI and tractography. It is located immediately posterior to the central sulcus and around the intraparietal sulcus; and connects the superior and inferior parts of the parietal cortex. The location and the trajectory of SIPS are consistent with those observed in post-mortem fibre dissection studies by Sachs (1892) and Vergani et al. (2014). SIPS was identified consistently across a large number of subjects from three independent dMRI datasets, and the existence of the tract was further corroborated by statistical evidence. These findings place SIPS in a good position to channel neuronal communication between the distant cortical areas underlying visuo-vestibular integration necessary for optic-flow processing and perception of self-motion. In vivo identification and characterisation of SIPS using dMRI data and tractography will open new avenues to further studying this tract in relation to diseases, development and brain functions.

## Electronic supplementary material

Below is the link to the electronic supplementary material.

Supplementary material 1 (DOCX 2289 kb)
