# Supplementary material for: Computational neuroanatomy of human stratum proprium of interparietal sulcus
Source: Brain Struct Funct. 2017 Sep 4;223(1):489–507. doi: 10.1007/s00429-017-1492-1 (PMC5772143; doi:10.1007/s00429-017-1492-1)
Supplement: Supplementary file 1 — Supplementary material 1 (DOCX 2289 kb) [file 429_2017_1492_MOESM1_ESM.docx]

Supplementary information for

Computational neuroanatomy of human stratum proprium of interparietal sulcus

Maiko Uesaki^1,2,3 *^, Hiromasa Takemura^2,4,5 *^ and Hiroshi Ashida^1^

* : corresponding authors

Author institutions:

1. Department of Psychology, Graduate School of Letters, Kyoto University, Kyoto, Japan
2. Japan Society for the Promotion of Science, Tokyo, Japan
3. Open Innovation & Collaboration Research Organization, Ritsumeikan University, Osaka, Japan
4. Center for Information and Neural Networks (CiNet), National Institute of Information and Communications Technology, and Osaka University, Osaka, Japan
5. Graduate School of Frontier Biosciences, Osaka University, Osaka, Japan

Contact information:

Maiko Uesaki

Open Innovation & Collaboration Research Organization, Ritsumeikan University, Osaka, Japan

uesaki@gst.ritsumei.ac.jp

Hiromasa Takemura

Center for Information and Neural Networks (CiNet), National Institute of Information and Communications Technology, and Osaka University, Osaka, Japan

htakemur@nict.go.jp

# **Author contribution:**

Designed the study: MU HA. Performed the experiments: MU HA. Analysed the data: MU HT. Contributed analysis tools: HT. Wrote the paper: MU HT HA.


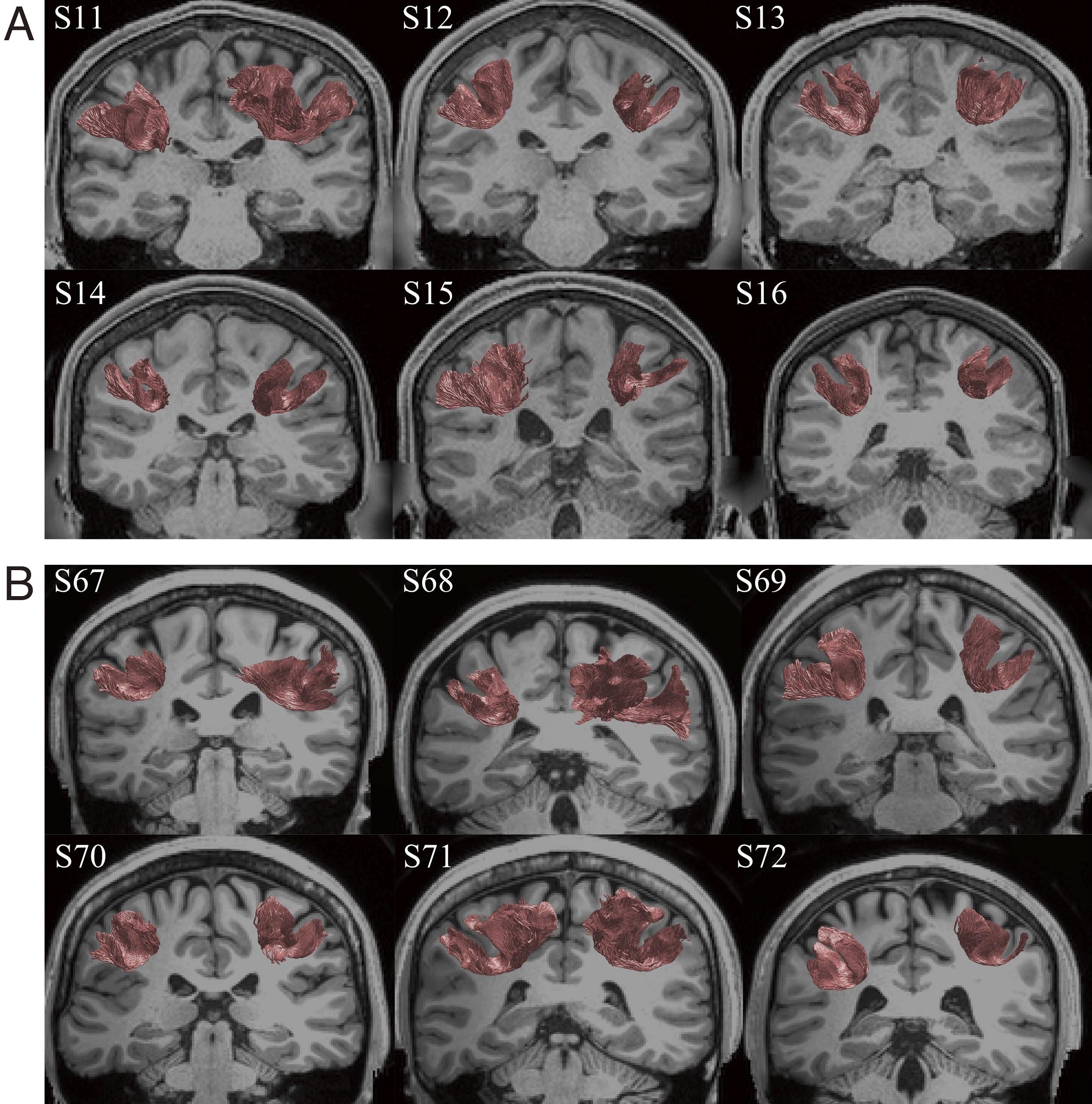


**Supplementary Figure 1.** SIPS identified in representative subjects from WU-Minn HCP (**A.** S11-S16) and MGH-USC HCP (**B.** S67-S72) datasets. Conventions are identical to those in Figures 1 and 2.


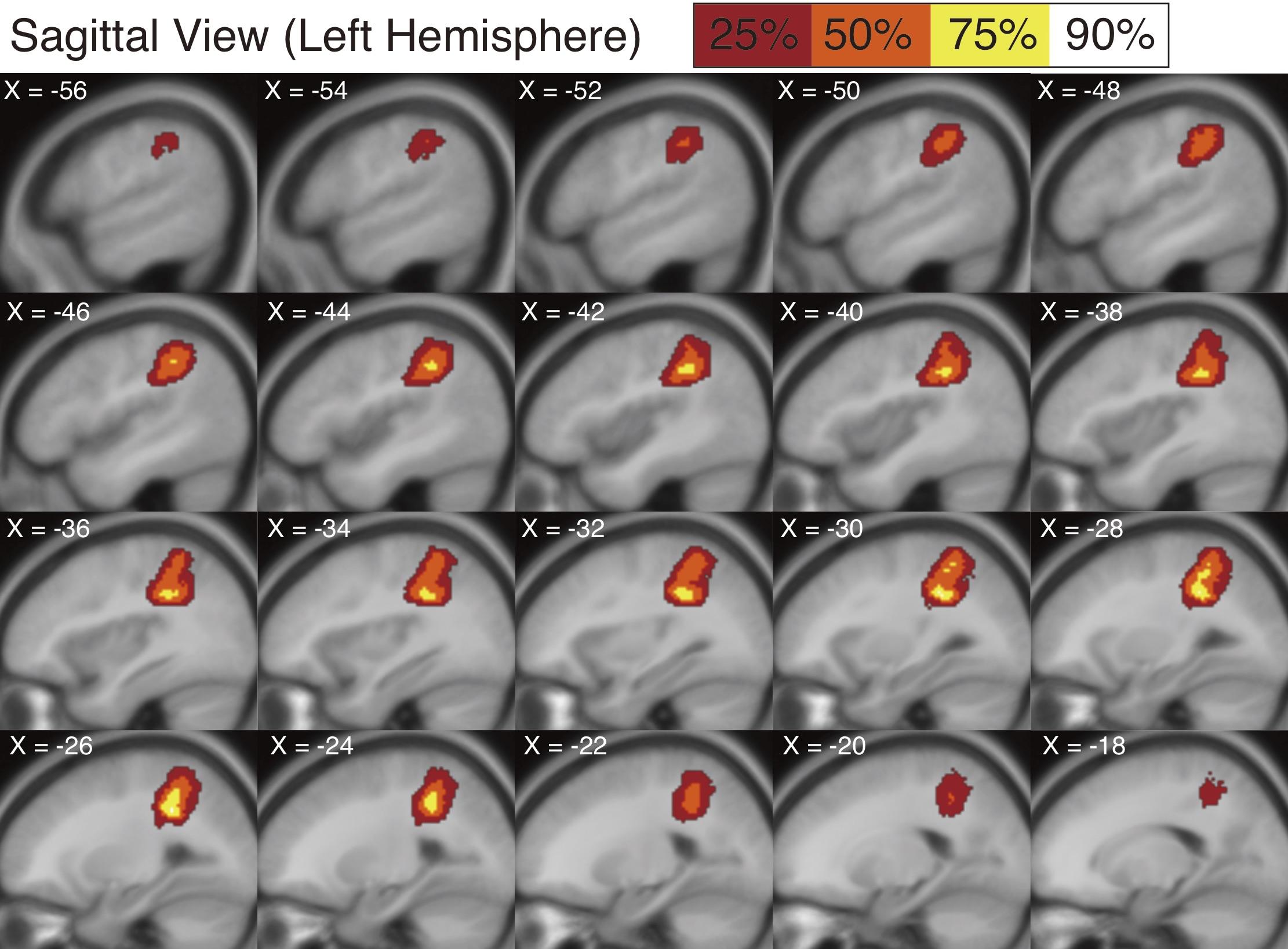


**Supplementary Figure 2.** Probabilistic population average of 100 subjects in MNI152 space, as shown in sagittal slices (left hemisphere). Colour coding indicates the degree of overlap across subjects. See Materials and methods in the main text for the methods used to create the population atlas.


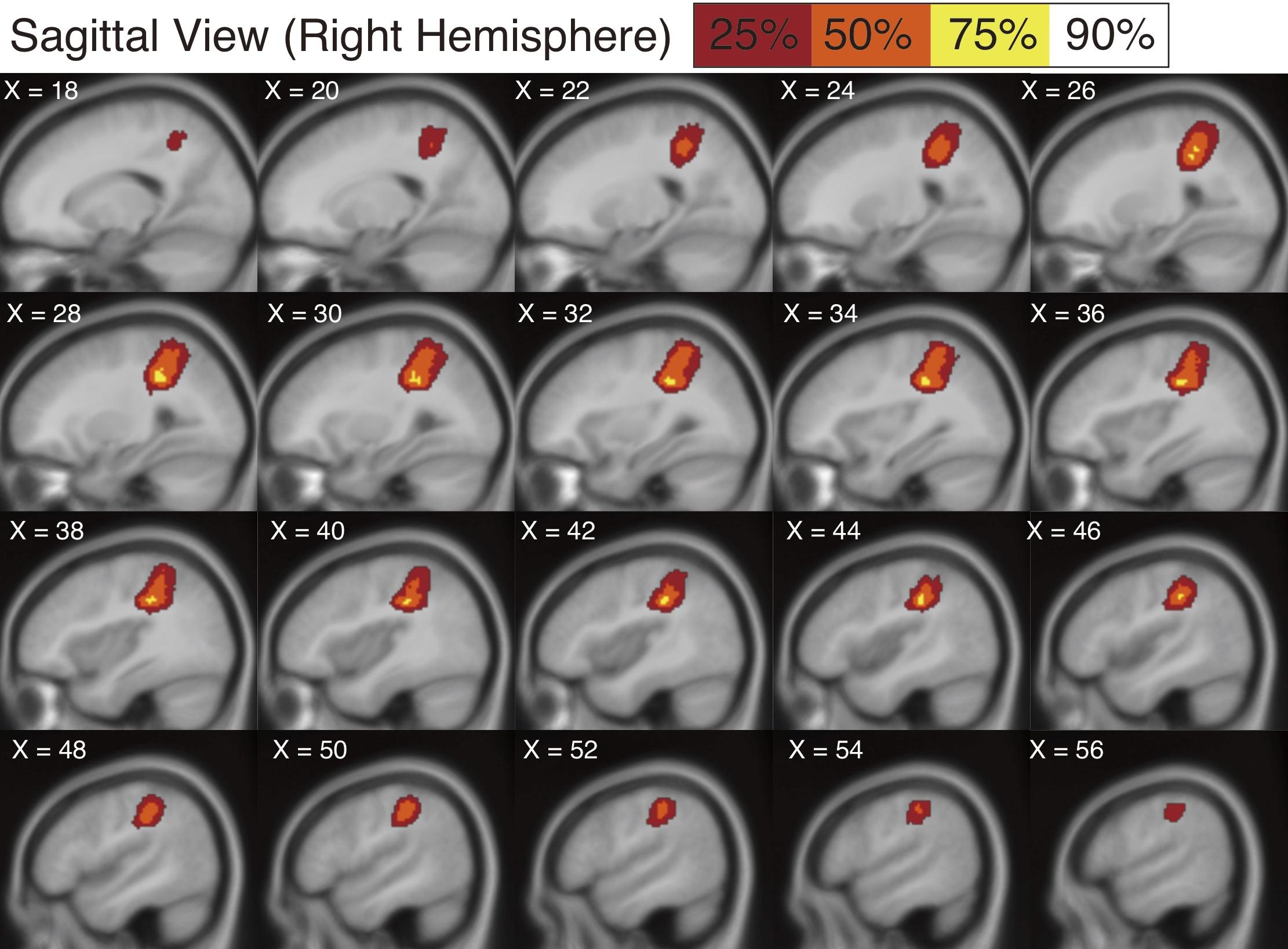


**Supplementary Figure 3.** Probabilistic population average of 100 subjects in MNI152 space, as shown in sagittal slices (right hemisphere). Colour coding indicates the degree of overlap across subjects. See Materials and methods in the main text for methods used to create the population atlas.


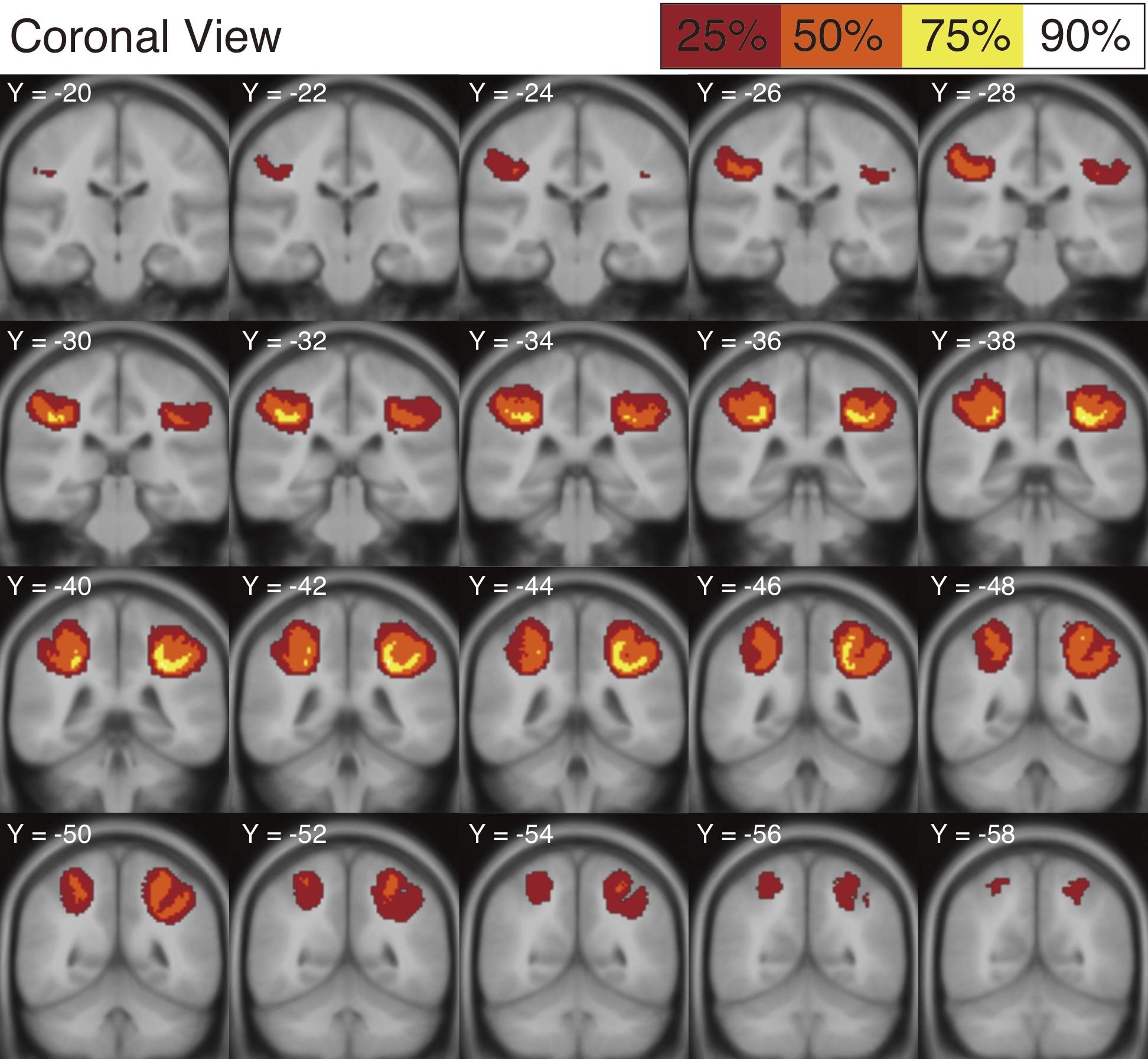


**Supplementary Figure 4.** Probabilistic population average of 100 subjects in MNI152 space, as shown in coronal slices. Colour coding indicates the degree of overlap across subjects. See Materials and methods in the main text for methods used to create the population atlas.


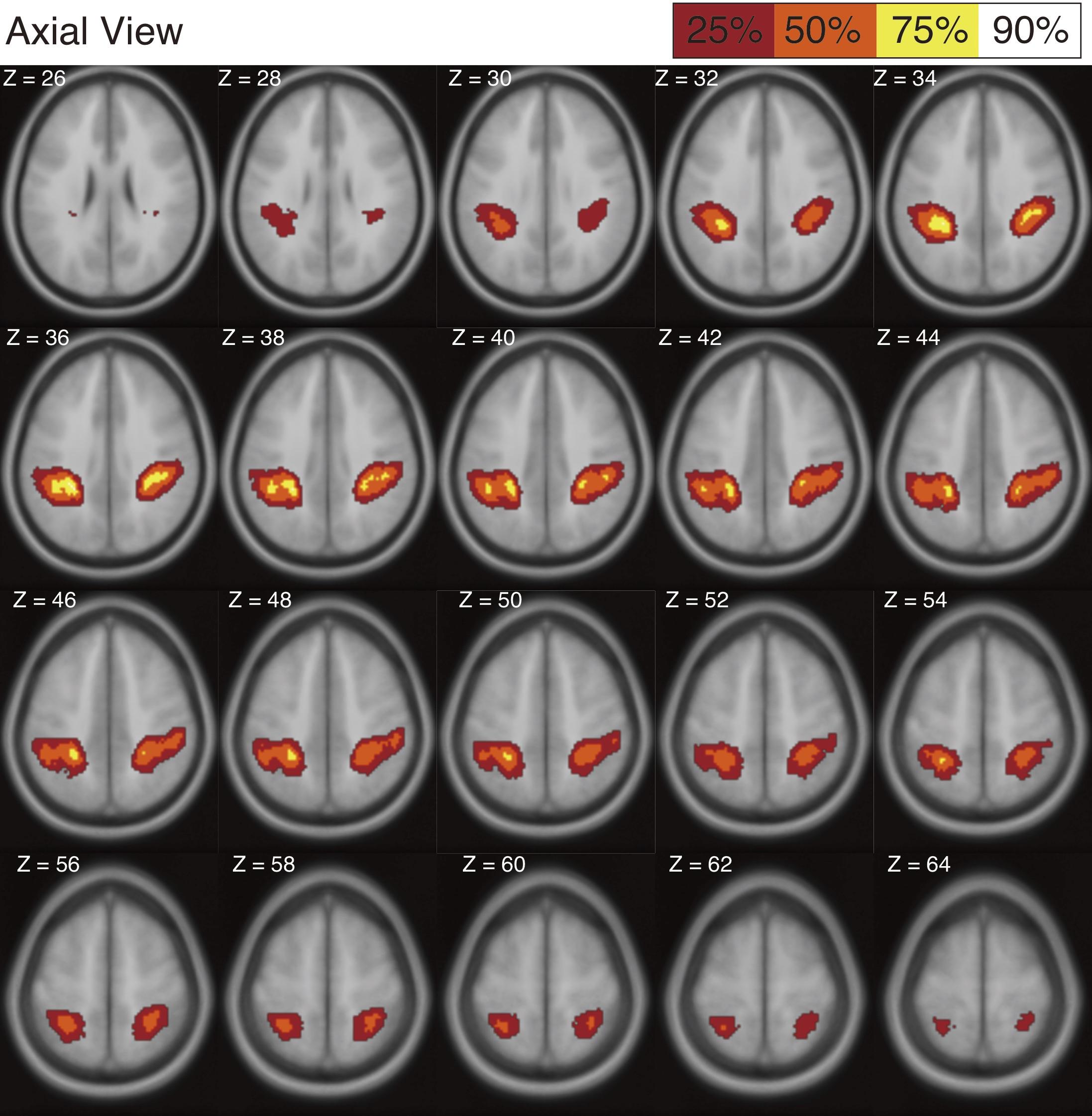


**Supplementary Figure 5.** Probabilistic population average of 100 subjects in MNI152 space, as shown in axial slices. Colour coding indicates the degree of overlap across subjects. See Materials and methods in the main text for methods used to create the population atlas.


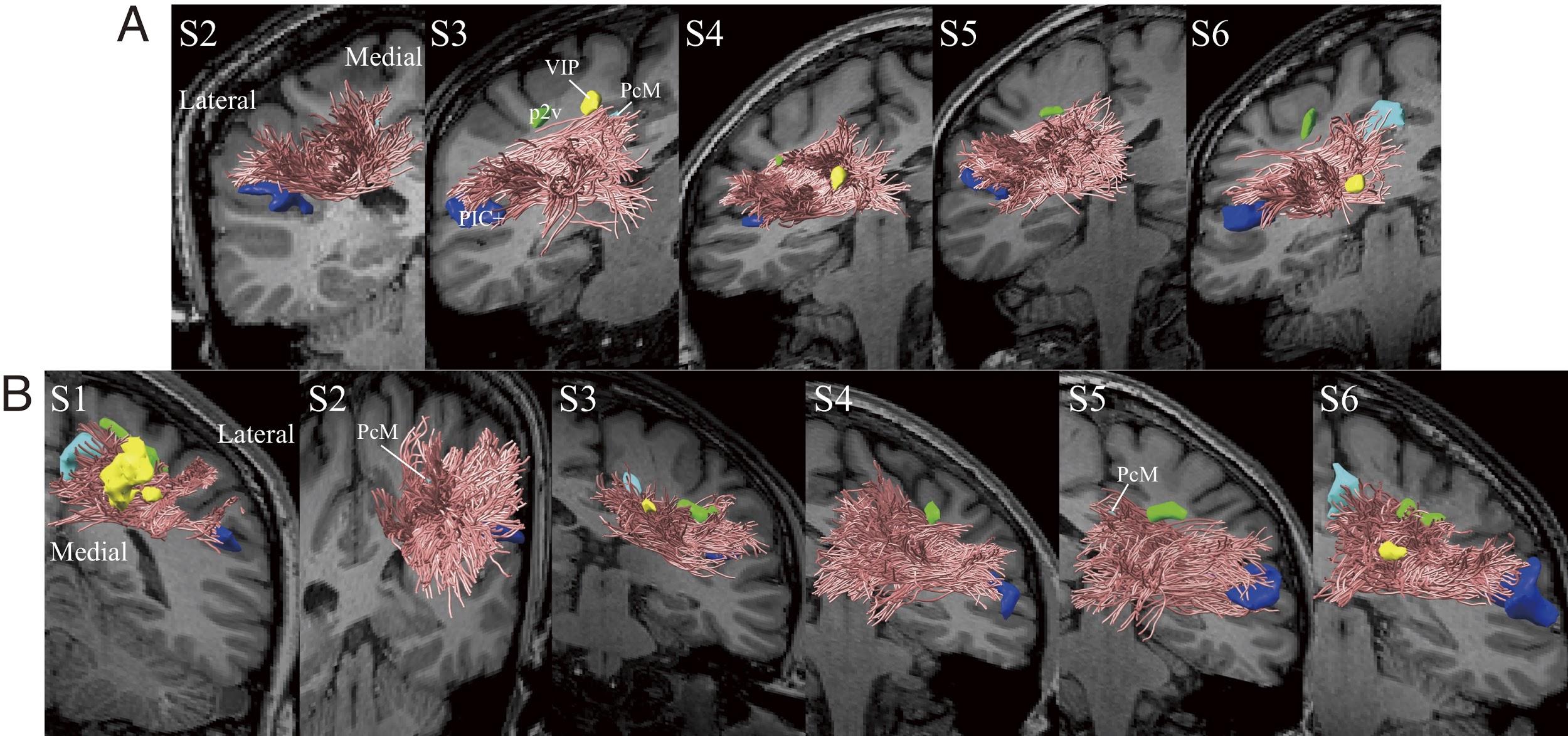


**Supplementary Figure 6.** Spatial proximity between SIPS endpoints and optic-flow selective cortical ROIs in remaining 11 hemispheres (A. left hemispheres; B. right hemispheres). While the ventro-lateral endpoints of SIPS are consistently located adjacent to PIC+, there is some variability in the spatial relations between the parietal ROIs (PcM, VIP and p2v) and the dorso-medial SIPS endpoints across hemispheres. Conventions are identical to those in Figure 7.
